# Drug effects on metabolic profiles of *Schistosoma mansoni* adult male parasites detected by ^1^H-NMR spectroscopy

**DOI:** 10.1371/journal.pntd.0008767

**Published:** 2020-10-12

**Authors:** Alessandra Guidi, Greta Petrella, Valentina Fustaino, Fulvio Saccoccia, Sara Lentini, Roberto Gimmelli, Giulia Di Pietro, Alberto Bresciani, Daniel Oscar Cicero, Giovina Ruberti

**Affiliations:** 1 Institute of Biochemistry and Cell Biology, National Research Council, Campus A. Buzzati-Traverso, Monterotondo (Rome) Italy; 2 Department of Chemical Science and Technologies, University of Rome Tor Vergata, Rome, Italy; 3 Department of Translational Biology, IRBM Science Park Spa, Pomezia (Rome), Italy; University of Calgary, CANADA

## Abstract

Schistosomiasis is one of the most devastating neglected tropical parasitic diseases caused by trematodes of the genus *Schistosoma*. Praziquantel (PZQ) is today the only drug used in humans and animals for the treatment of schistosomiasis but unfortunately it is poorly effective on larval and juvenile stages of the parasite. Therefore, it is urgent the discovery of new drug targets and compounds. We have recently showed that the anti-anginal drug perhexiline maleate (PHX) is very active on multiple developmental stages of *Schistosoma mansoni in vitro*. It is well known that PHX impacts the lipid metabolism in mammals, but the final target on schistosomes still remains unknown. The aim of this study was to evaluate the ability of ^1^H nuclear magnetic resonance (NMR) spectroscopy in revealing metabolic perturbations due to PHX treatment of *S*. *mansoni* adult male worms. The effects of PHX were compared with the ones induced by vehicle and gambogic acid, in order to detect different metabolic profiles and specificity of the PHX action. Remarkably a list of metabolites associated to PHX-treatment was identified with enrichment in several connected metabolic pathways including also the Kennedy pathway mediating the glycerophospholipid metabolism. Our study represents the first ^1^H-NMR metabolomic approach to characterize the response of *S*. *mansoni* to drug treatment. The obtained “metabolic fingerprint” associated to PHX treatment could represent a strategy for displaying cellular metabolic changes for any given drug and to compare compounds targeting similar or distinct biochemical pathways.

## Author summary

Schistosomiasis is a chronic and debilitating neglected tropical parasitic disease caused by the helminth *Schistosoma*. The control and treatment of the disease rely almost exclusively on praziquantel (PZQ), poorly effective on some developmental stage of the parasite. Identification of novel targets and drugs is required. The aim of this study was to use a ^1^H-NMR metabolomic approach to characterize the response of *Schistosoma mansoni* to perhexiline maleate (PHX), a multi-stage schistosomicidal drug previously investigated by our group. Remarkably we identified a metabolic signature specifically associated to drug-treatment in adult male parasites. This approach could contribute to the identification of novel targets and biochemical pathways implicated in parasite development and survival.

## Introduction

*Schistosoma* is the causing agent of one of the most devastating parasitic neglected disease, the schistosomiasis. More than 200 million people are infected and 800 million are at risk of infection in endemic countries. The three main species infecting humans are *S*. *haematobium*, *S*. *japonicum*, and *S*. *mansoni* [1]. The cure against schistosomiasis relies on chemotherapy with praziquantel (PZQ). PZQ is the only drug currently available to treat infected people, but unfortunately it is poorly effective on immature and juvenile parasites [2]. The constant and massive use of a single drug has raised concerns about the possibility of emerging drug resistance. With this prospective the discovery and development of drugs to be used as alternative, and/or in combination with PZQ appears mandatory.

We recently discovered multiple series of compounds able to kill the parasite *in vitro* [3,4]. Among them we showed that the drug perhexiline maleate (PHX) is active *in vitro* on larval (schistosomula), juvenile, and adult parasites [3]. In humans PHX has proven to be effective in patients with refractory angina [5]. In addition, it was shown to improve myocardial energetics and function in chronic cardiac failure and symptomatic hypertrophic cardiomyopathy [6,7]. PHX, still used in Australia and New Zealand, was withdrawn from the market in several countries due to toxic effects observed after chronic treatment in poor metabolizer individuals due to polymorphisms of the cytochrome P450 2D6 (CYP2D6) enzyme [8]. PHX is thought to be an inhibitor of carnitine palmitoyl transferase enzymes, namely CPT-1 and CPT-2 and impact long-chain fatty acid metabolism [9,10]. In contrast to previous findings, recent advances in *Schistosoma* metabolism showed that this parasite lacks CPT-1 and CPT-2 [11–13] and that fatty acid oxidation still remains controversial in schistosomes [11,14], therefore the mechanism of action of PHX in this parasite is yet to be clarified.

Metabolomics is a powerful technique, which simultaneously detects metabolites in biological fluids, cells, tissues, and whole organisms to obtain information about metabolic processes at baseline and treated conditions. Liquid Chromatography Mass Spectrometry (LC-MS) and ^1^H Nuclear Magnetic Resonance (^1^H-NMR) are the main tools. While the former is able to quantify metabolites even at very low concentrations by extracting them via liquid chromatography, the latter is suitable for untargeted qualitative and quantitative analysis of metabolites in complex mixture without extraction [15]. NMR based metabolomics has successfully been applied to characterize the metabolite composition of parasites and other human pathogens [16,17].

Metabolomics in the field of parasitosis is a remarkable tool of investigation to shed light onto inner metabolism, host–parasite interaction [18], and ultimately to identify essential metabolic pathways of the parasites that can be targeted for therapeutic intervention [19, 20]. Metabolomic analysis of treated parasites has proven effective to the identification of drug-induced perturbations to specific parasite metabolites and pathways [21–23].

In the present study, for the first time, we set out to use ^1^H NMR spectroscopy to analyze the metabolic status of S. *mansoni* and to identify potential treatment-associated signatures. We examined the metabolic changes that occur within time when *S*. *mansoni* adult male parasites are treated with vehicle or two different concentrations of PHX or gambogic acid (GA). The levels of some metabolites and the enrichment of some metabolic pathways associated to PHX or GA treatment allowed to differentiate the effects of the two compounds.

This study provides new, to our knowledge, insights for displaying cellular metabolic changes in *S*. *mansoni* for a given drug and comparing compounds targeting similar or distinct biochemical pathways.

## Methods

### Reagents

Chemical reagents, Dimethyl sulphoxide (DMSO), fetal bovine serum (FBS), gambogic acid (GA), perhexiline maleate (PHX) were purchased from Sigma-Aldrich (Saint Louis, USA); Dulbecco-Modified Eagle’s Medium (DMEM), HEPES, L-glutamine from Lonza (Basel, Switzerland); antibiotic-antimycotic reagent (100×) from Thermo Fisher Scientific (Waltham, USA).

### Ethical statement

Animal work was approved by the National Research Council animal welfare committee (OPBA) and by the competent authorities of the Italian Ministry of Health (authorizations no. 25/2014-PR and no. 336/2018-PR). All experiments were conducted in respect to the 3R rules according to the ethical and safety rules and guidelines for the use of animals in biomedical research provided by the relevant national and international laws.

### Maintenance of the *S*. *mansoni* life cycle

A Puerto Rican strain of *S*. *mansoni* was maintained by passage through albino *Biomphalaria glabrata*, as the intermediate host, and ICR (CD-1) outbred female mice as definitive host as previously described [24]. Female 4- to 7-week-old mice (Envigo, Udine, Italy) were housed with the following conditions: 22°C, 65% relative humidity, 12/12 h light/dark photocycle, standard food and water ad libitum Mice were infected using 150–200 single sex male cercariae by the tail immersion technique. Adult parasites were harvested from mice 7–8 weeks after infection by reversed perfusion of the hepatic portal system and mesenteric veins and cultured in DMEM complete tissue culture medium.

### Parasites culture

*S*. *mansoni* adult male worms, isolated from infected mice, were cultured in complete DMEM tissue culture medium and allowed to recover for 24 hours at 37°C in 5% CO_2_ atmosphere. ^1^H-NMR can detect metabolic changes associated within a shorter time frame, so that the status of parasites viability should be compatible with the analysis. Therefore, the worms were treated with vehicle (DMSO), PHX (5 μM, 10 μM), or GA (0.25 μM, 1 μM) for short time: compound concentrations were chosen according to what previously observed in terms of parasites viability [3]. The experiments were repeated at least 2 times and a triplicate of each experimental condition was collected to test the reproducibility and to reduce technical bias. Viability of *S*. *mansoni* adult male worms was assessed under a Leica MZ12 stereomicroscope by a multiple phenotypical score as previously reported [3,4] resulting 100% at 6 hours in all experimental conditions and between 50–80% at 24 hours (50% at the highest concentration and 70–80% at the lower concentration of both PHX and GA).

### Preparation of parasites extracts

In order to evaluate the minimum number of parasites to consistently detect a reasonable number of metabolites, 10, 25, 50, and 90 worms were employed for metabolites extraction. Following spectra analysis, the sample of 25 parasites showed to give appropriate spectra in terms of quality and reproducibility. Therefore, for all compound treatments, 75 parasites/sample were placed in 90 mm dish culture plates with 20 ml of DMEM complete medium. Six and 24 hours after drug exposure parasites were harvested, divided into 25 worms per sample (technical replicate), washed extensively with saline solution, and the pellets stored a -80°C until metabolites extraction. Three technical replicates, for at least two biological experiments, were processed.

Metabolites extraction protocol from whole parasites was adapted from tissue extraction techniques previously described [25]. In particular, metabolites from adult male worms frozen at -80°C for at least 1 hour were extracted using 0.5 mL of chloroform/methanol/water extraction buffer at the final ratio of 1:3:1. The extraction was performed comparing three different disruption methods: glass tissue Dounce homogenizer, electronic pestle, and TissueLyser machinery (Retsch, Qiagen). Using both glass tissue Dounce homogenizer and electronic pestle, worms were manually homogenized in the extraction buffer while with the TissueLyser automated method, worms were disrupted by rapid agitations (30 Hz) in the presence of a 3 mm metal bead. Agitations were performed twice for 2 minutes and samples frozen in liquid N_2_ prior each agitation. At the end of each extraction method, sample were put on a laboratory shaker featuring a horizontal (left to right) linear action to provide vigorous shaking for 1 hour at 4° C in a final volume of 1 mL of extraction buffer. Finally, all extract mixtures were centrifuged (1000 x g, 5 min, 4°C), supernatants collected, dried under vacuum conditions, and stored at -80°C until NMR analyses.

### NMR sample preparation

Worm extracts were dissolved in 100 μL of phosphate buffer 50 mM pH 7.4 in D2O solvent, with the addition of trimethylsilylpropionic acid (TSP) (0.5 mM) as internal standard solution. After centrifugation (2 sec, 14.000 x g, 4°C), a volume of 50 μL of this solution was inserted in a 1.7 mm NMR tube.

### NMR acquisition experiment

All ^1^H-NMR experiments were performed at 25°C on Bruker Avance 600 MHz equipped with a triple resonance 1.7 mm TXI probe and a SampleJet autosampler, using a noesypr1d (1D Nuclear Overhauser effect spectroscopy with water pre-saturation) pulse sequence with acquisition time of 2 s, relaxation delay of 3 s, 4096 transients, 4 dummy scans, tm = 100 ms, spectral width of 20 ppm, for a total acquisition time of 6 hours.

### Metabolite identification and quantification

Resonance assignments and quantifications were performed using Chenomx Suite 8.5 that provides a comprehensive database of metabolites, which can be used for manual deconvolution. The acquired spectra were processed using 0.5 Hz of line broadening followed by manual phase and baseline corrections, and in some cases automatic shim correction. A total of 43 metabolites were identified and quantified. Concentration sums of ADP+ATP and glucose+glucose-1-phosphate+glucose-6-phosphate were also considered. Chemical shifts were corroborated using bidimensional spectra, like ^1^H-^1^H TOCSY (total correlated spectroscopy), ^1^H-^13^C and ^1^H-^31^P HSQC (heteronuclear single quantum coherence). For a detailed table and experimental conditions see Supplementary Information (S1 Table).

### Statistical analysis

Multivariate data analysis was carried out using SIMCA-P (version 15.0.2. Umetrics AB, Umea, Sweden). All data were normalized, to account for different extraction efficiency, by using probabilistic quotients [26] and Unit-Variation scaled [27]. Classification models were constructed using Orthogonal PLS modeling (OPLS) [28], both in the discriminant (OPLS_DA) and numerical Y-variable (OPLSY) versions, as suitable. Metabolite concentrations measured using Chenomx were used as variables. The robustness of the models was evaluated by the following parameters: R^2^Y, predicted percentage of the response; R^2^X, variation of X explained by the model and Q^2^, goodness of prediction. R^2^ varies between 0 and 1, Q^2^ varies between -1 and 1. When Q^2^ value is higher than 0.5 the predicted model is good [29]. In the models, the influence on Y variation of every variable, called Variable Importance in the Projection (VIP), was used to considered which metabolites are involved in every supervised analysis. Moreover, Anova of the cross-validated residual (CV ANOVA) tests were performed to assess the significance of multivariate models. All these parameters were calculated using SIMCA.

Heatmap was drawn by R ggplot2 [30] applying the complete linkage method to find similar clusters within metabolites and treatment conditions. The log2 fold change was based on the normalized metabolite concentrations derived from Chenomx

### Network analysis

Network analysis was performed in Cytoscape [31]. In particular, networks were built by MetScape app [32] in the context of human metabolic networks, using a database developed by extracting and integrating information from KEGG COMPOUND Database and Edinburgh Human Metabolic Network (EHMN).

MetScape builds a network from a list of query compounds and extends the connections also to compounds that are taking part in the reactions in which query compounds are involved.

### Metabolic pathways enrichment

MSEA was performed using MetaboAnalyst pathway analysis tool [33]. In detail, we performed the hypergeometric test using the KEGG metabolic pathway library annotated for *S*. *mansoni*. The reference was based on the *S*. *mansoni* metabolome and pathways were selected according to p-value < 0.05 and impact > 0.3.

## Results

### Metabolite extraction and ^1^H-NMR analysis

In order to investigate the effect of PHX-treatment on the metabolic profile of *S*. *mansoni* adult male worms, we first assayed different disruption methods (electronic pestle, glass tissue Dounce homogenizer, and TissueLyser machinery) to obtain ^1^H-NMR spectra with sufficiently good signal to noise allowing the quantification of the highest number of metabolites. We found that the glass tissue Dounce homogenizer and TissueLyser led to the identification of essentially the same number of metabolites, even though the latter gave a higher concentration thus allowing us to reduce the number of parasites up to 25 worms/sample. Since parasites preparation is a labor-intensive procedure, the reduction of the number of parasites is advantageous especially in terms of number of host animals (mice) to be used in these studies.

**Fig 1:**
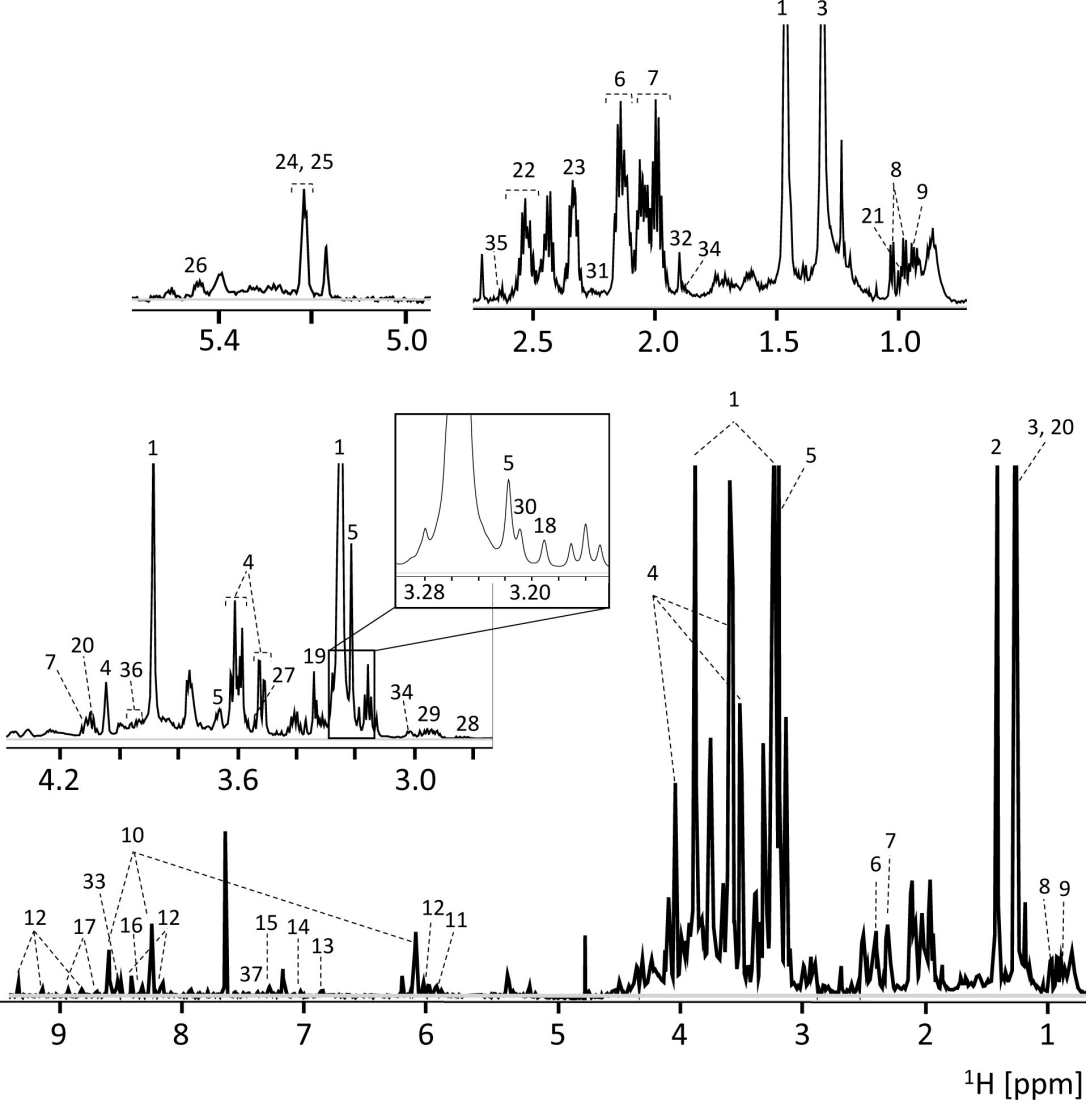
Typical 600 MHz 1H-NMR spectrum of Schistosoma extract obtained in H_2_O. 1: Betaine; 2: Alanine; 3: Threonine; 4: myo-Inositol; 5: sn-Glycero-3-phosphocholine (3GPC); 6: Glutamine; 7: Proline; 8: Valine; 9: Isoleucine; 10: AMP; 11: UDP-N_Acetylglucosamine; 12: NAD+; 13: Tyrosine; 14: Histidine; 15: Tryptophan; 16: Adenosine; 17: Niacinamide; 18: Choline; 19: Methanol; 20: Lactate; 21: Leucine; 22: Gluthatione; 23: Glutamate; 24: Glucose; 25: Glucose-1-phosphate; 26: Glucose-6-phosphate; 27: Glycine; 28: Aspartate; 29: Asparagine; 30: O-Phosphocholine; 31: Succinate; 32: Acetate; 33: ADP+ATP; 34: Lysine; 35: Methionine; 36: Serine; 37: Phenylalanine.

A representative ^1^H-NMR spectrum of the extracts obtained from *S*. *mansoni* worms is shown in Fig 1. The spectra are very information-rich and complex. Forty-three compounds were unequivocally assigned, quantified, and grouped into metabolic pathways or chemical classes such as redox system, lipid, and energy metabolisms including amino acids, intermediary metabolites, soluble phospholipids, and purine and pyrimidine nucleotides (Table 1 and S2 Table).

**Table 1:** Metabolites identified in *S*. *mansoni* worm extracts and classified according to their biological role.

Amino Acids	Aerobic and anaerobic respiration
Alanine	Acetate
Asparagine	Adenosine
Aspartate	ADP
Glutamate	AMP
Glutamine	ATP
Glycine	Formate
Histidine	Glucose
Isoleucine	Glucose-1-Phosphate
Leucine	Glucose-6- Phosphate
Lysine	Guanosine
Methionine	Inosine
Phenylalanine	Lactate
Proline	NAD+
Serine	Succinate
Threonine	UDP-N-acetylglucosamine
Tryptophan	UMP
Tyrosine	**Lipid Metabolism**
Valine	Choline
**Oxidative stress**	Phosphocholine (PC)
Glutathione (GSH)	3-Glycerophosphocholine (3-GPC)
Niacinamide	**Osmolytes**
**Alcohols**	Betaine
Methanol	myo-Inositol
	Taurine

### PHX-treatment associates with variations in the metabolite profile of *S*. *mansoni* adult male worms

In order to identify metabolomic signatures associated to PHX treatment, two concentrations of PHX (5 and 10 μM) previously shown to impair viability [3] were used to treat *S*. *mansoni* adult male worms *in vitro*. GA (0.25 and 1 μM) was used as reference compound to evaluate the specificity of the PHX associated variations with respect to generalized metabolic changes possibly due to toxic effects of a compound. The concentrations of PHX and GA were chosen in order to affect viability to a similar phenotypical extent. The viability was assessed by a multiple parameter score system as previously described [3,4]. The parasites used for the experiments, while damaged, were viable and the score never declined below 50%. Each compound treatment was investigated at two time points (6 and 24 hours).

**Fig 2:**
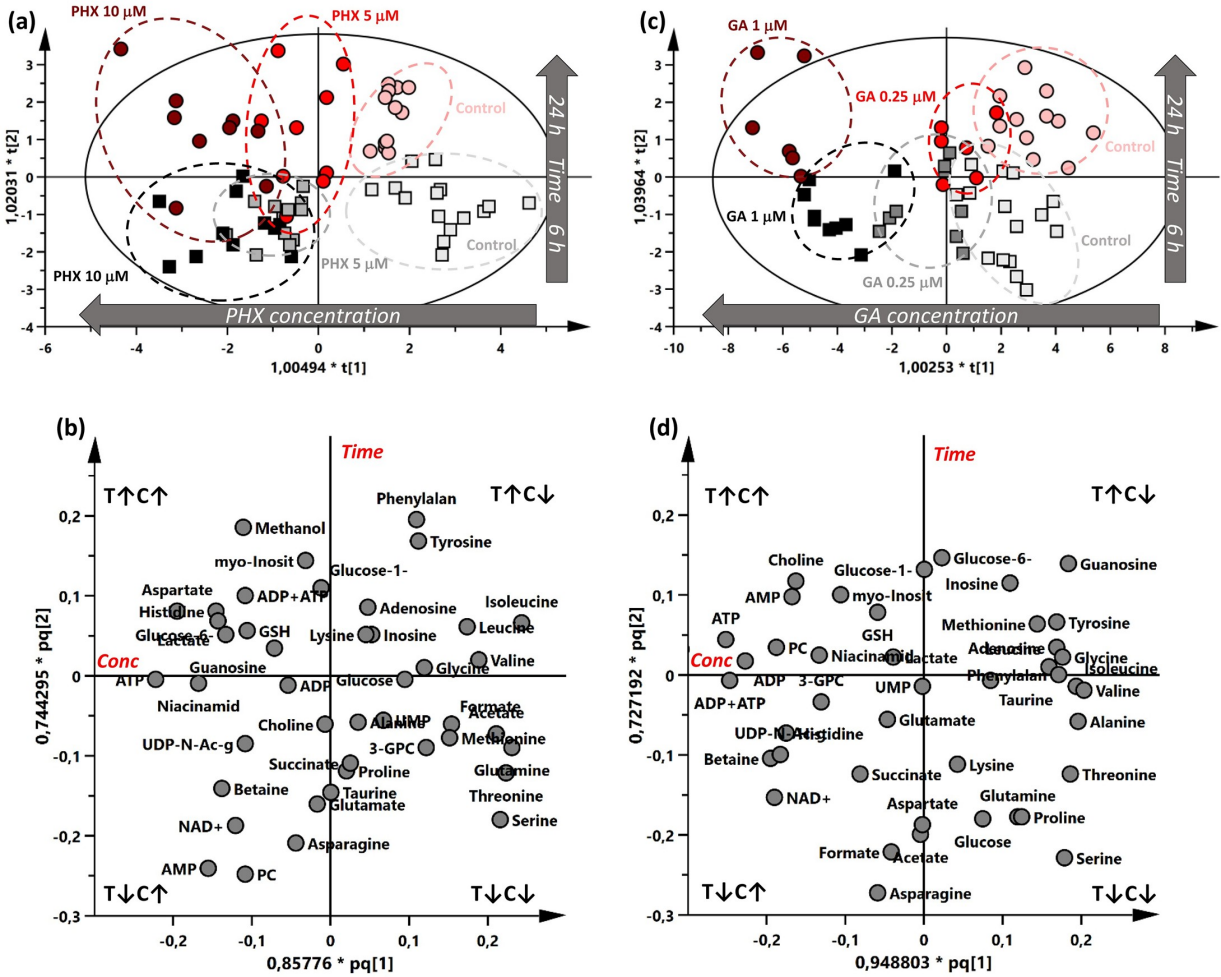
OPLSY score and loading plots of PHX and GA treatments. (a,b) PHX treatment (N: 68; A: 2+5+0; R^2^X: 0.717; R^2^Y: 0.733; Q^2^: 0.439; CV Anova: 1.3E-05); (c,d) GA treatment (N:57; A: 2+3+0; R^2^X: 0.757; R^2^Y: 0.728; Q^2^: 0.619; CV Anova: 3.5E-03). Boxes and circles represent samples collected after 6 h and 24 h respectively. Experiments were repeated 2–5 times, with three technical replicates each. GA3-GPC: 3-glycerophosphocholine; Glucose-6: glucose-6-phosphate; Glucose-1: glucose-1-phosphate; GSH: glutathione; PC: phosphocholine; Phenilalan: Phenylalanine; UDP-N-Ac-g: UDP-N-acetylglucosamine.

In each experiment, metabolites were extracted and quantified by ^1^H-NMR. The variation of metabolite concentrations was analyzed using an orthogonal partial least squares (OPLS) Y model [28], in which the effects of time and drug concentrations were evaluated along two orthogonal axes (Fig 2). For this analysis we considered in addition to the concentration of 43 metabolites, also the sum of ADP and ATP.

The best metabolic profile separation was obtained between worms treated with 10 μM PHX or vehicle at both time points whereas the separation was less pronounced at 5 μM PHX both at 6 h and 24 h treatment (Fig 2A).

With regard to the GA-treated samples, a marked metabolic separation was seen at 1 μM at both time points, while the lowest concentration did not seem to produce clearly distinct profiles (Fig 2C).

The corresponding loading plots show the variation of metabolites concentration upon PHX (Fig 2B) and GA (Fig 2D) treatments linking these differences to both time and drug concentration exposure. Metabolites, which lie on the two main diagonals, are ascribed to be modulated by both time and concentration. For example, AMP, PC, NAD+, phenylalanine, tyrosine, serine and choline concentrations were perturbed both by time and PHX concentration (Fig 2B). AMP, serine, guanosine and NAD+ showed the same behavior after GA treatment (Fig 2D).

Since 10 μM PHX and 1 μM GA, both at 6 and 24 h, yielded a clear perturbation of the metabolic profile, they represent the most reasonable experimental conditions to reveal a possible effect on parasite metabolome due either PHX or GA treatment.

### Comparison of metabolites levels altered by either PHX or GA treatments

**Fig 3:**
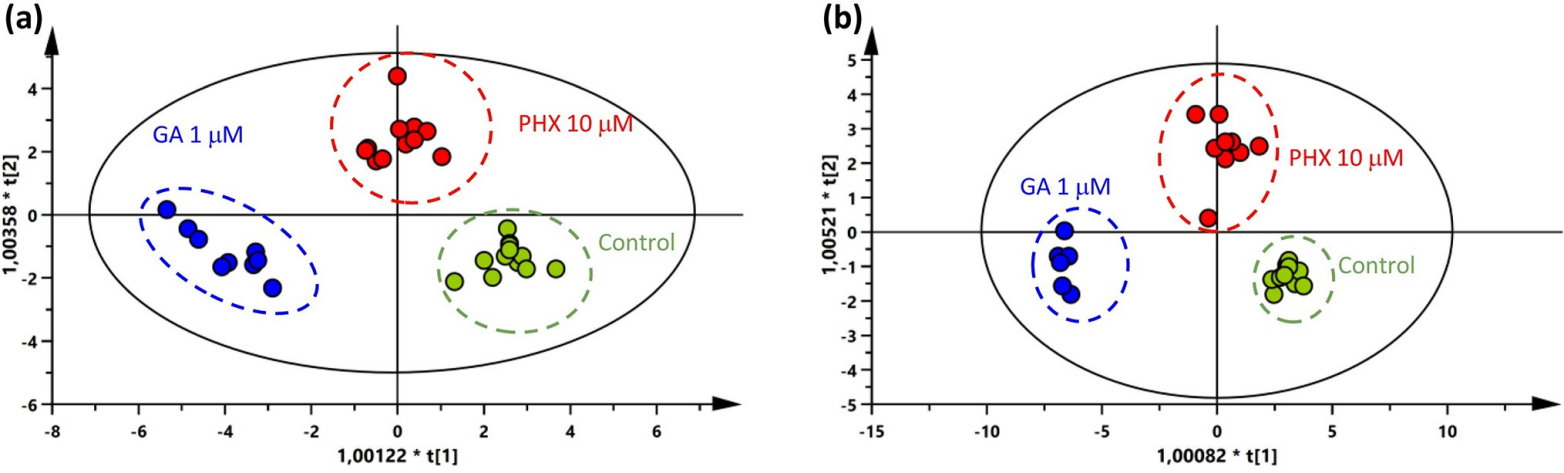
OPLS-DA score plots of PHX (10 μM) and GA (1μM) treated-samples. (a) 6 h treatment; N: 33; A: 2+4+0; R^2^X: 0.773; R^2^Y: 0.913; Q^2^: 0.857; CV Anova: 4.9E-08 and (b) 24 h treatment; N: 27; A: 2+3+0; R^2^X: 0.725; R^2^Y: 0.934; Q^2^: 0.857; CV Anova: 6.3E-10. Red and blue circles are samples treated with PHX and GA respectively and green circles are vehicle-treated samples (controls).

To compare the effects of PHX and GA on metabolite concentrations, a multivariate discriminant analysis (OPLS-DA) [24] among compound-treated samples and controls was performed. To this end we also considered the sum of glucose, glucose 1-phosphate and glucose 6-phosphate as an extra variable. Metabolic profiles of samples treated with PHX and GA were clearly and significantly different from each other with respect to control samples both at 6 and 24 h (Fig 3).

**Fig 4:**
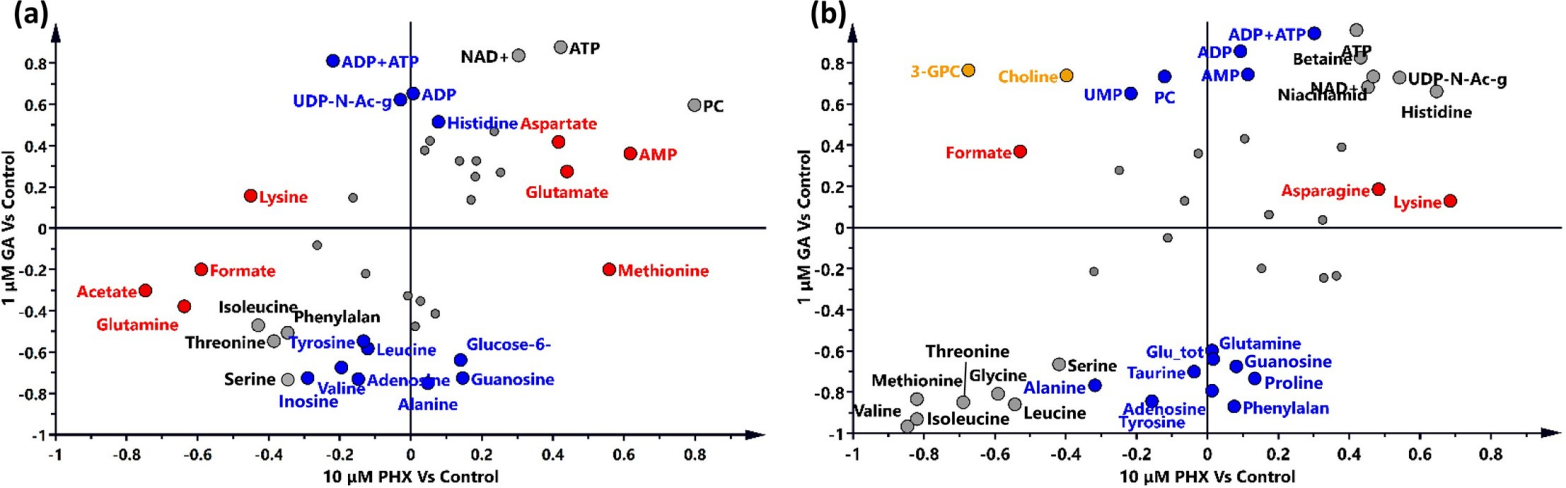
SUS-plots of PHX and GA treatments. (a) Samples were treated with the maximum concentration of PHX and GA, respectively 10 μM and 1 μM, for (a) 6h and (b) 24h. Metabolite concentrations showing VIP>1 altered by GA, PHX, or both compounds are showed in blue, red, and grey. Concentrations of choline and 3-glycerophosphocholine (marked in yellow) show opposite behavior in GA and PHX treatment. Abbreviations are the same of Fig 2; Glu tot: concentration sum of glucose, glucose-1-phosphate and glucose-6-phosphate.

In order to investigate which pattern of metabolites was perturbed after 6 and 24 h compound treatment four supervised models were applied (S1 and S2 Figs). Metabolic fingerprints of samples treated with the maximum concentrations of GA and PHX after 6 and 24 h appeared significantly different from controls (S1 and S2 Figs). In order to understand which metabolic pattern was characteristic of PHX and GA, the respective loadings were compared using shared and unique structure plots (SUS-plots, Fig 4).

We observed that the concentration levels of 12 metabolites resulted selectively altered in samples treated for 6 h with GA, whereas 8 were modulated in PHX-treated samples. Seven metabolites were found to change irrespective of the PHX or GA treatment (Fig 4A). Upon 24 h treatment, the magnitude of the metabolite variations was amplified with 14 resulting altered with both PHX and GA while 3 were PHX specific and 14 GA specific (Fig 4B).

**Fig 5:**
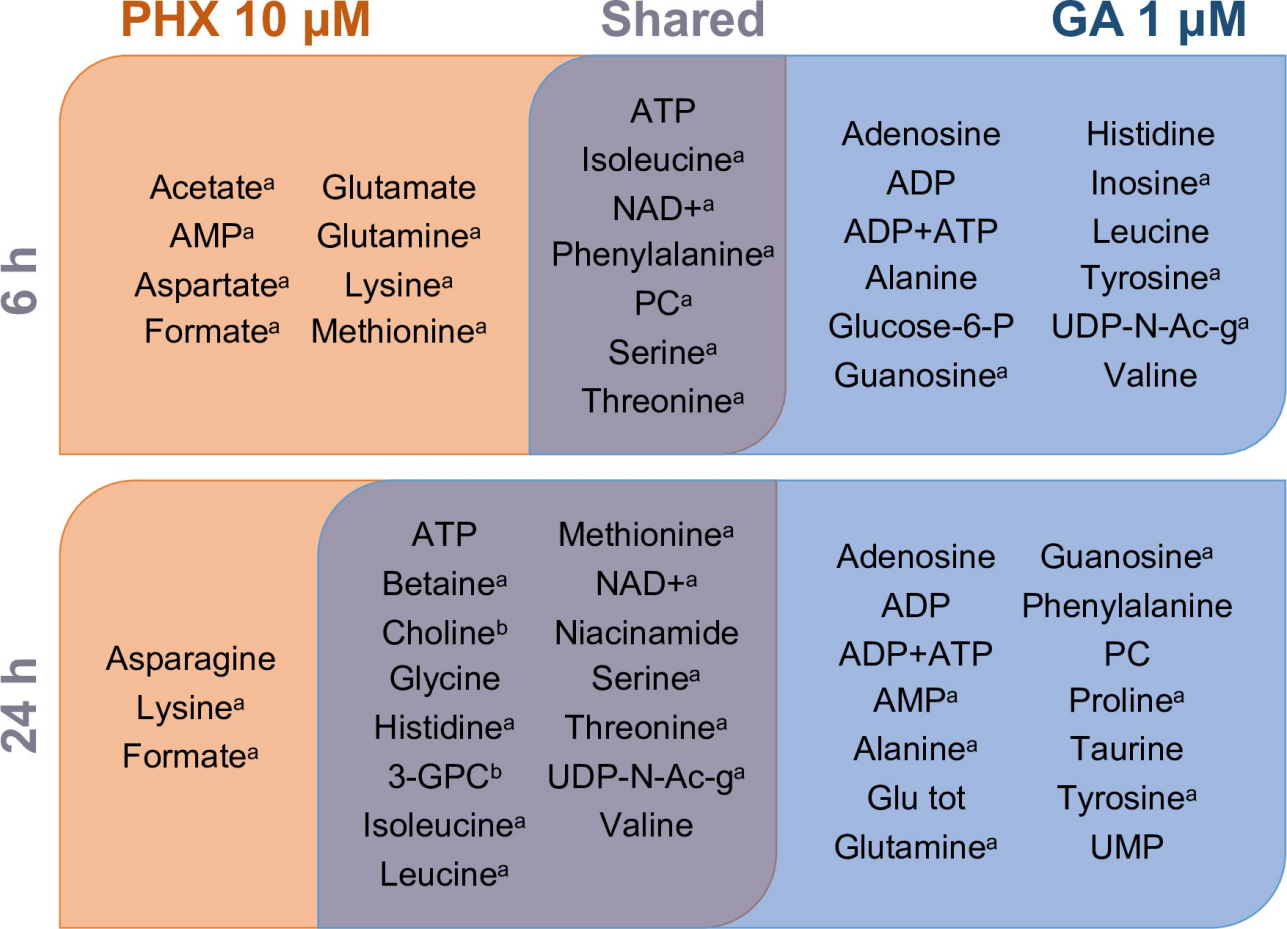
Summary of metabolites altered in all treatment conditions. a) Metabolites modulated by both time of exposure and drug concentration upon treatment perturbed (time x concentration); b) metabolites whose concentration show opposite behavior in PHX- and GA-treated samples. Abbreviations are the same of Fig 2.

Interestingly, the levels of choline and 3-glycerophosphocholine varied in an opposite way between PHX- and GA-treated samples at 24 h, suggesting that metabolic pathways regulation of these metabolites are differently impacted by the two treatments (Fig 4B). A complete list of the metabolite changes, with respect to treatment observed is summarized in Fig 5.

**Fig 6:**
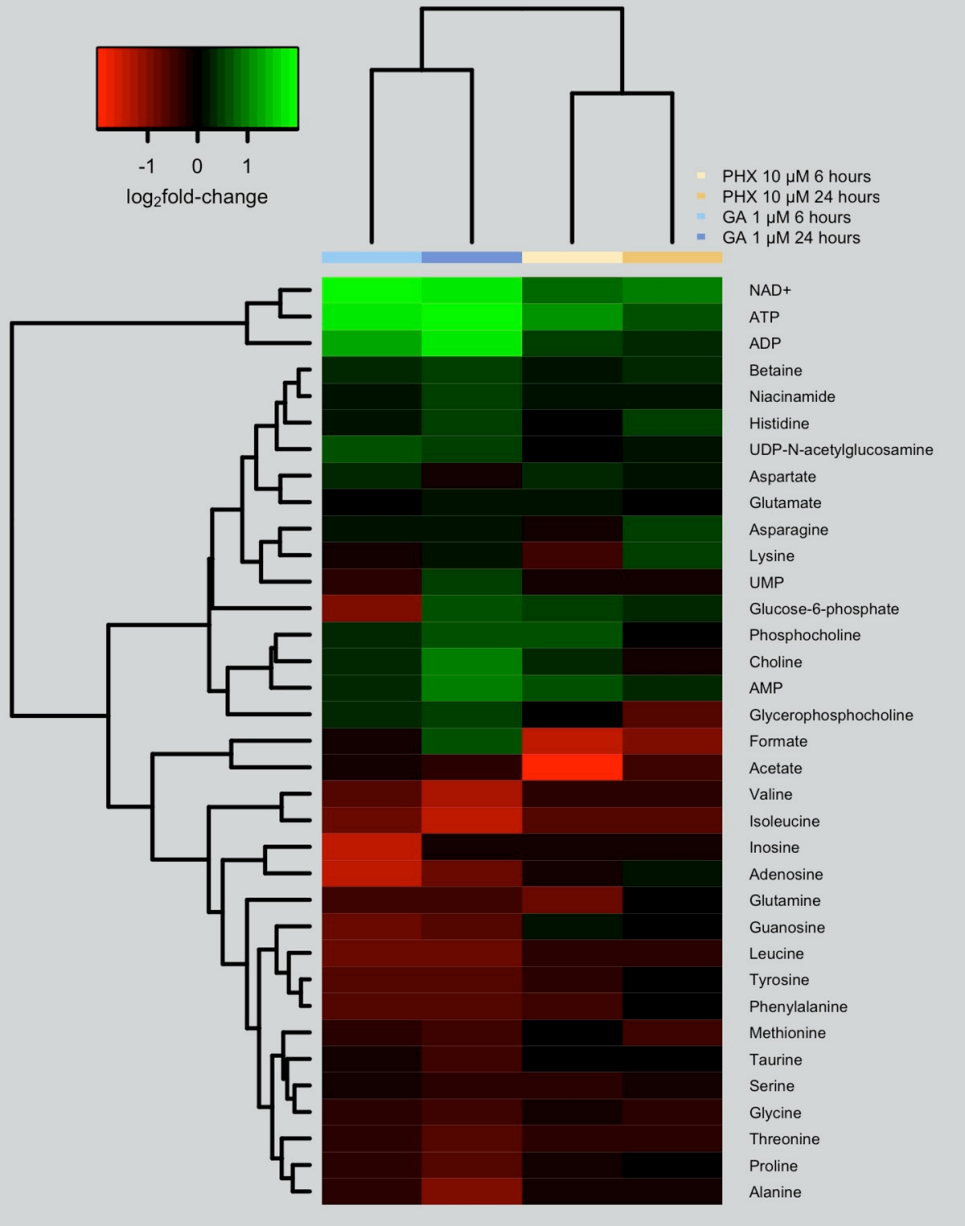
Heatmap and biclustering of treatment conditions and modulated metabolites. Data are represented as log_2_ fold-changes using time-specific vehicle as reference.

The 35 metabolites emerged from the discriminant analysis (Fig 4) represent the metabolites globally modulated upon compound treatment (Fig 5). The fold-changes of these metabolites, with respect to the vehicle treatment, were used to cluster samples based on time and drug treatment in an unsupervised analysis. Remarkably, PHX and GA treatments belong to two separate clusters, indicating that metabolic profiles are specifically impacted by treatments (Fig 6).

### Metabolic pathway analysis

To gain insight into pathways possibly modulated upon PHX and GA treatments we applied a network analysis. To this aim metabolites modulated by each treatment and involved in a common reaction were linked in order to identify those metabolites whose modulation is highly related upon treatment. The approach was based on the assumption that the specific effect of a treatment might impact metabolites residing in close proximity and participating to common metabolic axis.

**Fig 7:**
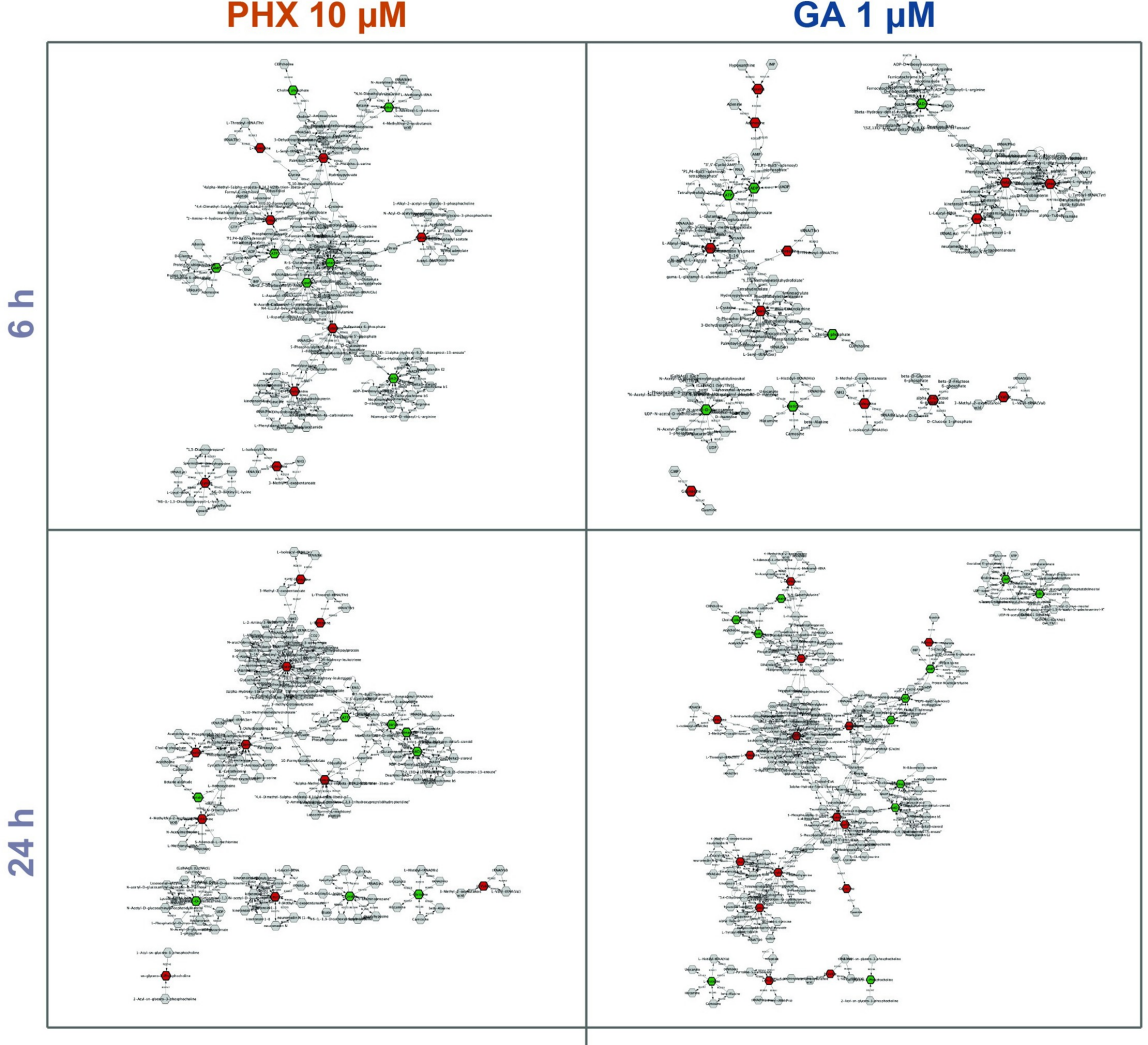
Neighbor-extended networks of metabolites impacted by PHX and GA treatments. Green and red dots represent respectively an increase or a decrease of the metabolite level in compound-treated samples. Gray nodes represent “neighbor” metabolites.

Starting from the list of metabolites modulated by PHX and GA (hereafter “seed metabolites”) a network was built by linking in “connected components” also metabolites participating to the same biochemical reaction not present in the seed metabolites list (Fig 7 and S3 Fig). The networks shown were built based on the annotations in the MetScape library for *Homo sapiens* biochemical pathways.

The small number of specific modulated metabolites makes the identification of pathways associated to a specific compound-treatment not an easy task. For these reasons we widened the networks by adding the “neighbor metabolites”, i.e. metabolites which were not detected by ^1^H-NMR, but that are part of the same reaction in which the seed metabolites participate (Fig 7). Pathways are generally seen as a series of connected reactions, which finally flow up either to a branch point or to a final metabolite, which cannot be further metabolized. In this view, any pathway ultimately stems from a unique pathway-substrate, in the form already proposed by Newsholme & Crabtree [34]:

S→A→B→C→D→…→P

with S, pathway-substrate; A to D, intermediate metabolites; P, final product or branch point. Hence, by considering two of our seeds (i.e. A and D), the network was extended to neighbor metabolites (one step forward, from A to B, and one step backward, from D to C) to finally get the missing links, which ultimately explain how a precise metabolic pathway can be affected. This approach, while undoubtedly an oversimplified view of the metabolic complexity, could overcome the limits due to: i) ^1^H-NMR sensitivity and ii) lack of accumulation of some of the neighbor metabolites. Then we analyzed the extended list of metabolites starting from the hypothesis that whenever the concentration of a metabolite is perturbed, its variation can also affect metabolites that are partners into a common reaction.

The size of our “neighbor-extended” networks ranges from 141 to 225 connected nodes including the majority of the seed metabolites (78% on average). Noteworthy, PHX treatment at 6 h showed the highest number of connected seed metabolites (87%) (Fig 7). Similar results were obtained for PHX 5 μΜ (S3 Fig). These results suggest that seed metabolites, which were not directly connected (S3 Fig, left panel), are indeed in close proximity and therefore potentially involved in common metabolic processes (S3 Fig, right panel).

In order to identify pathways possibly impacted by PHX and GA treatments, we performed a Metabolite Set Enrichment Analysis (MSEA) using the metabolite lists of the expanded networks generated via MetScape (S3 Table). The library of metabolic pathways and the reference metabolome for the MSEA were specific for *S*. *mansoni* (see methods section for details). The results are shown in S4 Table and summarized in Table 2. A number of pathways resulted enriched by both PHX and GA treatments: aminoacyl-tRNA biosynthesis, arginine biosynthesis, glutathione metabolism, glycerophospholipid metabolism, glycine, serine and threonine metabolism, nicotinate and nicotinamide metabolism, and phenylalanine metabolism. Interestingly the concentration levels of choline and 3-GPC vary in an opposite direction in PHX- and GA-treated samples, suggesting that the biochemical pathway(s) in which these metabolites are embedded might be differentially perturbed by the two treatments. The GA treatment at 6 h resulted in the specific enrichment of D-glutamine and D-glutamate metabolism along with nitrogen metabolism, purine metabolism, starch and sucrose metabolism, and tyrosine metabolism. Pathways associated specifically to PHX treatment included: cysteine and methionine metabolism (6 h and 24 h), folate biosynthesis (6 h), glyoxylate, and dicarboxylate metabolism (6 h).

**Table 2:** Summary of metabolic pathways enriched in neighbor-extended networks.

Metabolic pathways*	PHX 10 μM		GA 1 μM	
**Time (h)**	**6**	**24**	**6**	**24**
Alanine, aspartate and glutamate metabolism	✓		✓	✓
Aminoacyl-tRNA biosynthesis	✓	✓		✓
Arginine biosynthesis		✓		✓
Cysteine and methionine metabolism	✓	✓		
D-Glutamine and D-glutamate metabolism			✓	
Folate biosynthesis	✓			
Glutathione metabolism	✓	✓		✓
**Glycerophospholipid metabolism**		✓		✓
**Glycine, serine and threonine metabolism**	✓	✓	✓	✓
Glyoxylate and dicarboxylate metabolism	✓			
Nicotinate and nicotinamide metabolism	✓	✓	✓	✓
Nitrogen metabolism			✓	
Phenylalanine metabolism	✓		✓	✓
Purine metabolism			✓	
Starch and sucrose metabolism			✓	
Tyrosine metabolism			✓	

*Metabolic pathways specific for PHX (orange) or GA (blue) treatment are color coded. Pathways involving choline and/or glycerophosphocholine are in bold.

**Fig 8:**
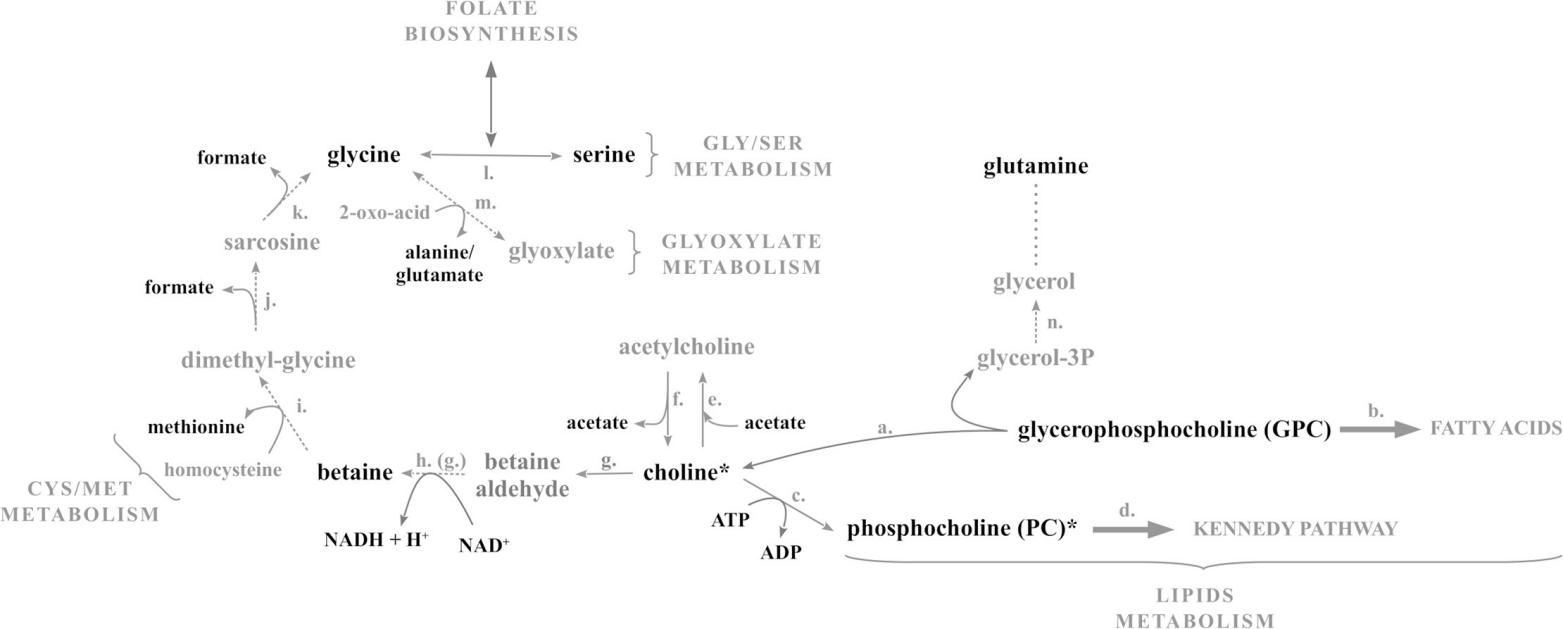
A scheme of putative connections of some metabolites impacted by PHX treatment and enriched PHX-associated metabolic pathways. Metabolites modulated by PHX are in bold and metabolites in close proximity, as inferred by extended pathway analysis, are in gray. This scheme is based on the nowadays knowledge of metabolic pathways of animals obtained from KEGG pathway (https://www.genome.jp/kegg/pathway.html), and/or MetaCyc (https://metacyc.org); accordingly to UniProt database). All *S*. *mansoni* enzymes are inferred by homology, with the exception of cholinesterase (f.) and choline oxidase/aldehyde dehydrogenase (g. and/or h.) activity that were experimentally demonstrated to be present in the parasite [35,36]. In addition, a glycerol/glutamine metabolic axis (n.) was experimentally demonstrated for *S*. *mansoni* [37]. a. Smp_054800, Smp_162910; b. Smp_025160, Smp_031190, Smp_166500, Smp_166530, Smp_171820; c. Smp_132570; d. Smp_124730; e. Smp_146910; g. Smp_094500, Smp_212180; h. methyltransferases; i. betaine-homocysteine S-methyltransferase; j. dimethyl-glycine dehydrogenase; k. sarcosine dehydrogenase; l. Smp_144570, Smp_179710; m. alanine-glyoxylate aminotransferase; n. glycerol kinase. 2-oxoacid refers to pyruvate or 2-oxoglutarate which react with glycine to give alanine or glutammate, respectively. * Highlights metabolites of the Kennedy pathway. Single-headed or double-headed arrows stand for alleged one-way or both-way reactions. Dotted lines indicate reactions inferred by analogy but not annotated in KEGG, while continuous lines indicate reactions with enzymes annotated in KEGG.

The majority of the PHX enriched pathways (Table 2) were represented in a unique scheme that summarizes the majority of PHX-associated metabolite variations observed in this study (Fig 8).

They are involved in the Kennedy pathway and in the betaine and folate metabolism, which in turn are linked together by the glycine-serine interconversion reaction. Side pathways like cysteine and methionine metabolism and, glyoxalate metabolism are also depicted (Fig 8). The central role of choline in bridging the glycine and serine metabolism to the glycerophospholipid metabolism is of note. In addition, its oxidation leads to betaine, a key osmolite and a methyl group donor in methylation (Fig 8).

## Discussion

Understanding how small molecules interfere with cellular metabolism is a critical part of modern drug development. Untargeted metabolomics offers a rapid and unbiased approach for the investigation of metabolic pathways, the discovery of drug modes of action, and of potential drug targets.

In this study, we applied for the first time a metabolomic analysis based on ^1^H-NMR spectroscopy on S. *mansoni* adult male worms treated with schistosomicidal compounds. As initial point, we focused our attention on setting the best technical procedure for the extraction of worm soluble metabolites. TissueLyser produced the best results in terms of qualitative and quantitative level of metabolite extraction and the minimum number of parasites required for the identification of a reproducible and accurate set of metabolites.

Using the ^1^H-NMR approach we succeeded into identifying 43 soluble metabolites of *S*. *mansoni* adult male worms. OPLS analysis allowed us to select the best experimental conditions to identify variation in metabolic profiles associated to PHX or GA treatments, thus demonstrating that the ^1^H-NMR approach could be effective in detecting metabolic perturbations induced by different compound treatments.

Finally, we attempted to ascribe metabolic pathways associated to parasite-treatments. Low-sensitivity is one of the drawbacks of the ^1^H-NMR technique; in addition, some metabolites might not accumulate in a pathway for the peculiar metabolic assets of a given species. Therefore, to overcome this limit, we took advantage of a neighbor-extended network analysis starting from the metabolites identified by ^1^H-NMR and building a network including the neighbor metabolites residing in their close proximity and participating to common metabolic axes.

The glycerophospholipids pathway is among the pathways enriched in PHX-treated samples and this was an intriguing, though not totally unexpected finding. Indeed, PHX has been largely recognized to perturb the lipid metabolism in mammals; and it seems that an effect on parasite’s lipids and choline metabolism could be also ascribed now to PHX. Importantly the lipid metabolism has for parasite biology a remarkable role, in particular for tegumental development and its turnover, and eggs production. Noteworthy, treatment with PHX or GA appears to modulate choline and 3-GPC in an opposite way: these metabolites were decreased in PHX-treated samples and increased in GA-treated ones, suggesting that the fate of both metabolites is differentially impacted by compound treatment.

In mammals, PHX is thought to impact the lipid metabolism by acting as inhibitors of CPT-1 and CPT-2 enzymes [8], whose role is to carry lipids inside mitochondrial for fatty acid oxidation [13]. While the genes encoding CPT-1 and -2 seem to be absent within the *Schistosoma* genome [11–13], in our previous study we demonstrated that female parasites treated with sub-lethal doses of PHX, showed accumulation of lipids within the vitellarium (lipid droplets) [3]. Importantly, the PHX treatment also decreases egg-laying *in vitro* in agreement with what previously reported on studies with the CPT-1 inhibitor etomoxir, that inhibited depletion of lipid reserves in fecund females worms *in vitro* and impaired schistosome egg production as well [14].

In the schematic representation of the PHX-associated enriched pathways where the metabolite variations observed in this study are highlighted (Fig 8), the choline has a central role. It acts as a hub bridging the lipid metabolism to glycine and serine metabolism, via betaine formation. As previously demonstrated adult male *S*. *mansoni* worms can exploit the oxidative route of choline bringing to betaine formation as well as to incorporate choline within PC, hence initiating the *de novo* synthesis of phosphatidylcholine *via* Kennedy pathway [36].

Within the mammalian host, adult *S*. *mansoni* couples reside in the mesenteric veins where male and female worms live paired and acquire nutrients directly from the blood of the host including among others carbohydrates, amino acids, lipids, and choline [38]. Parasite-derived lipids play important roles in: i) host-pathogen interactions and for their immunomodulatory properties which may contribute to the immune evasion mechanisms [39]; ii) the maintenance of surface integrity; iii) egg production; iv) cell-cell signaling [40]. Overall the lipid metabolism is of great interest in *S*. *mansoni* biology, mainly due to its role in development and survival of parasites; therefore, we concentrate our attention on the Kennedy pathway, relying on the uptake of exogenous choline into the cell for phosphatidylcholine synthesis. Choline is needed for the biosynthesis of phosphatidylcholine, the most predominant cell membrane phospholipid in *S*. *mansoni* cercariae, schistosomula, and adult worms [41], as a methyl-group donor, and for cholinergic neurotransmission. Choline is also metabolically interrelated to the folate pool and the cysteine and methionine metabolism, pathways both enriched in PHX-treated samples (Fig 8).

Overall the modulation of metabolism due to PHX treatment appears also to affect the ability of the parasite to utilize glutamine. Interestingly glutamine is amongst the most dysregulated metabolites detected in our analyses (Fig 6) and experimental evidences demonstrated that it can be used as precursor of many metabolites including glycerol supporting the existence of glyceroneogenesis in *S*. *mansoni* sporocysts [37]. Therefore, we can speculate that an increasing into mobilization and catabolism of GPC ensures the refueling of glycerol pool, whose levels may otherwise be lowered by lacking of synthesis from glutamine. It remains to be elucidated which, among the proposed pathways, is impacted by PHX treatment as an initiating event, and which one is impacted as a cascade effect.

Remarkably in protozoan parasites such as *Plasmodium falciparum* and *Trypanosoma brucei*, the Kennedy pathway has already been proposed as a potentially relevant drug target [42–48]. Further investigation of the Kennedy metabolic pathway in *S*. *mansoni* could be important for drug discovery and drug repositioning (i.e. PHX).

In conclusion our study represents the first ^1^H-NMR metabolomic approach to characterize the response of *S*. *mansoni* metabolome to a drug-treatment. The workflow proceeded through an optimization of sample preparation (parasites number, extraction protocol), ^1^H-NMR data acquisition, signal preprocessing, data analysis, and then to interpretation of the results. Multiple factors, such as dosage and exposure time, were extensively considered and tested in pilot studies before assessing the final conditions of both time points and drug concentration. The untargeted metabolic comparisons were always performed between two groups (PHX vs DMSO; GA vs DMSO; PHX vs GA) in order to extract useful information. The “metabolic fingerprints” associated to PHX or GA treatment *in vitro* were distinct and they could represent a strategy of displaying cellular metabolic changes for any given drug and to compare compounds targeting similar or distinct biochemical pathways.

## Supporting information

S1 Fig OPLS-DA score plots of GA-treated samples. (a) OPLS-DA score plots of GA (1μM) treated-sample vs control after 6 h N: 21; A: 1+2+0; R^2^X: 0.637; R^2^Y: 0.965; Q^2^: 0.923; CV Anova: 1.2E-06 and (b) 24 h N: 18; A: 1+1+0; R^2^X: 0.625; R^2^Y: 0.984; Q^2^: 0.970; CV Anova: 1.1E-09
(TIF)

S2 Fig OPLS-DA score plots of PHX-treated samples. (a) OPLS-DA score plots of PHX (10μM) treated-sample vs control after 6 h N: 24; A: 1+2+0; R^2^X: 0.477; R^2^Y: 0.960; Q^2^: 0.943; CV Anova: 2.2E-09 and (b) 24 h N: 21; A: 1+1+0; R^2^X: 0.339; R^2^Y: 0.938; Q^2^: 0.827; CV Anova: 2.5E-05
(TIF)

S3 Fig Pathway-networks of metabolites impacted by PHX and GA treatments. Networks are showed before (left panel) and after (right panel) neighbor-extension. Green and red colors represent respectively an increase or a decrease of the seed metabolite level in compound treated samples. Gray nodes represent “neighbor” metabolites.
(PDF)

S1 Table ^1^H, ^13^C and ^31^P assignments of metabolites identified in *Schistosoma* extract in H_2_O extracts. ^1^H and ^13^C chemical shifts are reported with respect to the TSP signal, and ^31^P chemical shifts are reported relative to 85% inorganic orthophosphoric acid.
(DOCX)

S2 Table Metabolite concentrations. (XLSX)

S3 Table Details on nodes of neighbor-extended networks. Metabolite annotations are reported according to MetScape library.
(XLSX)

S4 Table MSEA result tables. Enriched pathways of neighbor-extended networks upon PHX and GA treatment. Green: p-value < 0.05; Yellow: impact > 0.3
(XLSX)

## References

[1] McManus DP, Dunne DW, Sacko M, Utzinger J, Vennervald BJ, Zhou ZX. Schistosomiasis. Nat Rev Dis. Prim. 2018; 4: 11–19. 10.1038/s41572-018-0009-4 30093684

[2] Cioli D, Pica-Mattoccia L, Basso A, Guidi A. Schistosomiasis control: praziquantel forever? Mol Biochem Parasitol. 2014; 195: 23–29. 10.1016/j.molbiopara.2014.06.002 24955523

[3] Guidi A, Lalli C, Perlas E, Bolasco G, Nibbio M, Monteagudo E, et al Discovery and characterization of novel anti-schistosomal properties of the anti-anginal drug, Perhexiline and its impact on *Schistosoma mansoni* male and female reproductive systems. PLoS Negl Trop Dis. 2016; 10: e0004928 10.1371/journal.pntd.0004928 27518281 PMC4982595

[4] Guidi A, Lalli C, Gimmelli R, Nizi E, Andreini M, Gennari N, et al Discovery by organism based high-throughput screening of new multi-stage compounds affecting *Schistosoma mansoni* viability, egg formation and production. PLoS Negl Trop Dis. 2017; 11: e0005994 10.1371/journal.pntd.0005994 28985236 PMC5646872

[5] Cole PL, Beamer AD, McGowan N, Cantillon CO, Benfell K, Kelly RA, et al Efficacy and safety of perhexiline maleate in refractory angina. A double-blind placebo-controlled clinical trial of a novel antianginal agent. Circulation 1990; 81: 1260–1270. 10.1161/01.cir.81.4.1260 2180591

[6] Lee L, Campbell R, Scheuermann-Freestone M, Taylor R, Gunaruwan P, Williams L, et al Metabolic modulation with perhexiline in chronic heart failure: a randomized, controlled trial of short-term use of a novel treatment. Circulation 2005; 112: 3280–3288. 10.1161/CIRCULATIONAHA.105.551457 16301359

[7] Abozguia K, Elliott P, McKenna W, Phan TT, Nallur-Shivu G, Ahmed I, et al Metabolic modulator perhexiline corrects energy deficiency and improves exercise capacity in symptomatic hypertrophic cardiomyopathy. Circulation 2010; 122: 1562–1569. 10.1161/CIRCULATIONAHA.109.934059 20921440

[8] Ashrafian H, Horowitz JD, Frenneaux MP Perhexiline. Cardiovasc Drug Rev. 2007; 25: 76–97. 10.1111/j.1527-3466.2007.00006.x 17445089

[9] Kennedy JA, Unger SA, Horowitz JD. Inhibition of carnitine palmitoyltransferase-1 in rat heart and liver by perhexiline and amiodarone. Biochem Pharmacol. 1996; 52: 273–280. 10.1016/0006-2952(96)00204-3 8694852

[10] Kennedy JA, Kiosoglous AJ, Murphy GA, Pelle MA, Horowitz JD. Effect of perhexiline and oxfenicine on myocardial function and metabolism during low-flow ischemia/reperfusion in the isolated rat heart. J Cardiovasc Pharmacol. 2000; 36: 794–801. 10.1097/00005344-200012000-00016 11117381

[11] Bexkens ML, Mebius MM, Houweling M, Brouwers JF, Tielens AGM, van Hellemond JJ. *Schistosoma mansoni* does not and cannot oxidise fatty acids, but these are used for biosynthetic purposes instead. Int J Parasitol. 2019; 49: 647–656. 10.1016/j.ijpara.2019.03.005 31170410

[12] Berriman M, Haas BJ, LoVerde PT, Wilson RA, Dillon GP, Cerqueira GC, et al The genome of the blood fluke *Schistosoma mansoni*. Nature 2009; 460: 352–358. 10.1038/nature08160 19606141 PMC2756445

[13] Taylor CM, Wang Q, Rosa BA, Huang SC, Powell K, Schedl T, et al Discovery of anthelmintic drug targets and drugs using chokepoints in nematode metabolic pathways. PLoS Pathog. 2013; 9:e1003505 10.1371/journal.ppat.1003505 23935495 PMC3731235

[14] Huang SC, Freitas TC, Amiel E, Everts B, Pearce EL, Lok JB, et al Fatty acid oxidation is essential for egg production by the parasitic flatworm *Schistosoma mansoni*. PLoS Pathog. 2012; 8: e1002996 10.1371/journal.ppat.1002996 23133378 PMC3486914

[15] McKenzie JS, Donarski JA, Wilson JC, Charlton AJ. Analysis of complex mixtures using high-resolution nuclear magnetic resonance spectroscopy and chemometrics Prog Nucl Magn Reson Spectrosc. 2011; 59: 336–359. 10.1016/j.pnmrs.2011.04.003 22027342

[16] Giraudeau P. Challenges and perspectives in quantitative NMR. Magn Reson Chem. 2017; 55: 61–69. 10.1002/mrc.4475 27370178

[17] Markley JL, Brüschweiler R, Edison AS, Eghbalnia HR, Powers R, Raftery D, et al The future of NMR-based metabolomics. Curr Opin Biotech. 2017; 43: 34–40. 10.1016/j.copbio.2016.08.001 27580257 PMC5305426

[18] Kloehn J, Blume M, Cobbold SA, Saunders EC, Dagley MJ, McConville MJ. Using metabolomics to dissect host-parasite interactions. Curr Opin Microbiol. 2016; 32: 59–65. 10.1016/j.mib.2016.04.019 27200489

[19] Giannangelo CR, Ellis KM, Sexton AE, Stoessel D, Creek DJ. The role of metabolomics in antiparasitic drug discovery. In: Skype M, Cerdan R, Radulescu O, editors. Comprehensive analysis of parasite biology: from metabolism to drug discovery. Wiley-VCH Verlag GmbH & Co. KGaA 2016 pp. 321–341.

[20] Salinas JL, Kissinger JC, Jones DP, Galinski MR. Metabolomics in the fight against malaria. Mem Inst Oswaldo Cruz. 2014; 109: 589–597. 10.1590/0074-0276140043 25185001 PMC4156452

[21] Allman EL, Painter HJ, Samra J, Carrasquilla M, Llinas M. Metabolomic profiling of the Malaria box reveals antimalarial target pathways. Antimicrob Agents Chemother. 2016; 60: 6635–6649. 10.1128/AAC.01224-16 27572391 PMC5075069

[22] Vincent IM, Barrett MP. Metabolomic-based strategies for anti-parasite drug discovery. J Biomol Screen. 2015; 20: 44–55. 10.1177/1087057114551519 25281738

[23] Creek DJ, Barrett MP. Determination of antiprotozoal drug mechanisms by metabolomics approaches. Parasitology. 2014; 141:83–92. 10.1017/S0031182013000814 23734876 PMC3884841

[24] Lalli C, Guidi A, Gennari N, Altamura S, Bresciani A, Ruberti G. Development and validation of a luminescence-based, medium-throughput assay for drug screening in *Schistosoma mansoni*. PLoS Negl Trop Dis. 2015; 9:e0003484 10.1371/journal.pntd.0003484 25635836 PMC4312041

[25] Lin CY, Wu H, Tjeerdema RS, Viantb MR. Evaluation of metabolite extraction strategies from tissue samples using NMR metabolomics. Metabolomics 2007; 3: 55–67.

[26] Craig A, Cloarec O, Holmes E, Nicholson JK, Lindon JC. Scaling and normalization effects in NMR spectroscopic metabolomic data sets. Anal Chem. 2006; 78: 2262–2267. 10.1021/ac0519312 16579606

[27] van den Berg RA, Hoefsloot HC, Westerhuis JA, Smilde AK, van der Werf MJ. Centering, scaling, and transformations: improving the biological information content of metabolomics data. BMC Genomics. 2006; 7:142–156. 10.1186/1471-2164-7-142 16762068 PMC1534033

[28] Trygg J, Wold S. Orthogonal Projections to Latent Structures (OPLS). J Chemometr. 2002; 16: 119–128.

[29] Triba MN, Moyec LL, Amathieu R, Goossens C, Bouchemal N, Nahon P, et al PLS/OPLS models in metabolomics: the impact of permutation of dataset rows on the K-fold cross-validation quality parameters. Mol Biosyst. 2015; 11: 13–19. 10.1039/c4mb00414k 25382277

[30] Wickham H. ggplot2: Elegant graphics for data analysis. Springer-Verlag New York 2016 ISBN 978-3-319-24277-4. Available from: https://ggplot2.tidyverse.org.

[31] Shannon P, Markiel A, Ozier O, Baliga NS, Wang JT, Ramage D, et al Cytoscape: a software environment for integrated models of biomolecular interaction networks. Genome Res. 2003; 13: 2498–2504. 10.1101/gr.1239303 14597658 PMC403769

[32] Karnovsky A, Weymouth T, Hull T, Tarcea VG, Scardoni G, Laudanna C, et al Metscape 2 bioinformatics tool for the analysis and visualization of metabolomics and gene expression data. Bioinformatics 2012; 28: 373–80. 10.1093/bioinformatics/btr661 22135418 PMC3268237

[33] Xia J, Wishart DS, Valencia A. MetPA: a web-based metabolomics tool for pathway analysis and visualization. Bioinformatics 2011; 26: 2342–2344. 10.1093/bioinformatics/btq418 20628077

[34] Newsholme EA, Crabtree B. Theoretical principles in the approaches to control of metabolic pathways and their application to glycolysis in muscle. J Molec Cell Cardiol 1979; 11: 839–856. 490659 10.1016/0022-2828(79)90480-2

[35] Arnon R, Silman I, Tarrab-Hazdai R. Acetylcholinesterase of *Schistosoma mansoni* -functional correlates. Contributed in honor of professor Hans Neurath's 90th birthday. Protein Sci. 1999; 8: 2553–2561. 10.1110/ps.8.12.2553 10631970 PMC2144239

[36] Ancelin ML, Torpier G, Vial HJ, Capron A. Choline incorporation by *Schistosoma mansoni*: distribution of choline metabolites during development and after sexual differentiation. J Parasitol. 1987 73: 530–535. 3598803

[37] Khayath N, Mithieux G, Zitoun C, Coustau C, Vicogne J, Tielens AG, et al Glyceroneogenesis: an unexpected metabolic pathway for glutamine in *Schistosoma mansoni* sporocysts. Mol Biochem Parasitol. 2006;147:145–153. 10.1016/j.molbiopara.2006.02.002 16522333

[38] Young BW Podesta RB. Uptake and incorporation of choline by *Schistosoma mansoni* adults. Mol Biochem Parasitol. 1985;1 5:105–114. 10.1016/0166-6851(85)90112-4 4010702

[39] Giera M, Maria MM. Kaisar MMM, Derks RJE, Steenvoorden E, Kruize YCM, et al The *Schistosoma mansoni* lipidome: leads for immunomodulation. Anal Chim Acta 2018; 1037 107–118. 10.1016/j.aca.2017.11.058 30292284

[40] Furlong ST. Unique roles for lipids in *Schistosoma mansoni*. Parasitol Today 1991; 7: 59–62. 10.1016/0169-4758(91)90192-q 15463424

[41] Furlong ST, Caulfield JP. *Schistosoma mansoni*: sterol and phospholipid composition of cercariae, schistosomula, and adults. Exp Parasitol. 1988; 65: 222–231. 10.1016/0014-4894(88)90126-9 3350102

[42] Déchamps S, Wengelnik K, Berry-Sterkers L, Cerdan R, Vial HJ, Gannoun-Zaki L. The Kennedy phospholipid biosynthesis pathways are refractory to genetic disruption in *Plasmodium berghei* and therefore appear essential in blood stages. Mol Biochem Parasitol. 2010; 173: 69–80. 10.1016/j.molbiopara.2010.05.006 20478340

[43] Ancelin ML, Calas M, Vidal-Sailhan V, Herbute S, Ringwald P, Vial HJ. Potent inhibitors of *Plasmodium* phospholipid metabolism with a broad spectrum of in vitro antimalarial activities. Antimicrob Agents Chemother. 2003a; 47: 2590–2597. 10.1128/aac.47.8.2590-2597.2003 12878524 PMC166094

[44] Ancelin ML, Calas M, Bonhoure A, Herbute S, Vial HJ. In vivo antimalarial activities of mono- and bis quaternary ammonium salts interfering with *Plasmodium* phospholipid metabolism. Antimicrob Agents Chemother. 2003b; 47: 2598–2605. 10.1128/aac.47.8.2598-2605.2003 12878525 PMC166095

[45] Pessi G, Mamoun CB. Pathways for phosphatidylcholine biosynthesis: targets and strategies for antimalarial drugs, Future Lipidology 2006; 1: 173–180. 10.2217/17460875.1.2.173

[46] Biagiotti M, Dominguez S, Yamout N, Zufferey R. Lipidomics and anti- trypanosomatid chemotherapy. Clin Trans Med. 2017; 6: 27–37. 10.1186/s40169-017-0160-7 28766182 PMC5539062

[47] Gibellini F, Hunter WN, Smith TK. The ethanolamine branch of the Kennedy pathway is essential in the bloodstream form of *Trypanosoma brucei*. Mol Microbiol. 2009; 73: 826–843. 10.1111/j.1365-2958.2009.06764.x 19555461 PMC2784872

[48] Gibellini F, Smith TK. The Kennedy pathway. De novo synthesis of phosphatidylethanolamine and phosphatidylcholine. IUBMB Life 2010; 62: 414–428. 10.1002/iub.337 20503434

