# Supplementary figures and images for: Drug effects on metabolic profiles of Schistosoma mansoni adult male parasites detected by 1H-NMR spectroscopy
Source: PLoS Negl Trop Dis. 2020 Oct 12;14(10):e0008767. doi: 10.1371/journal.pntd.0008767 (PMC7580944; doi:10.1371/journal.pntd.0008767)

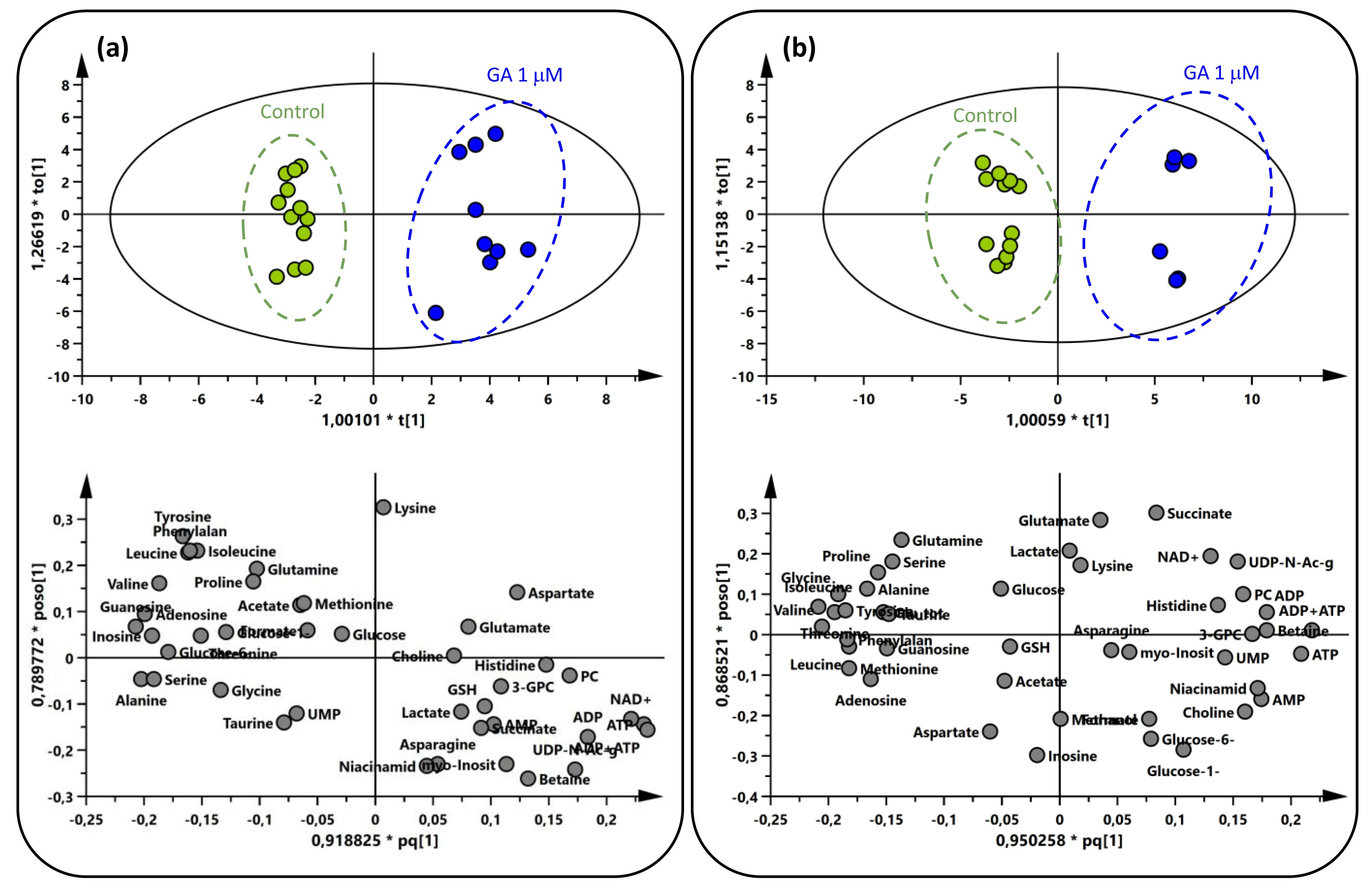

Supplement: S1 Fig — (a) OPLS-DA score plots of GA (1μM) treated-sample vs control after 6 h N: 21; A: 1+2+0; R2X: 0.637; R2Y: 0.965; Q2: 0.923; CV Anova: 1.2E-06 and (b) 24 h N: 18; A: 1+1+0; R2X: 0.625; R2Y: 0.984; Q2: 0.970; CV Anova: 1.1E-09 (TIF) [file pntd.0008767.s001.tif]

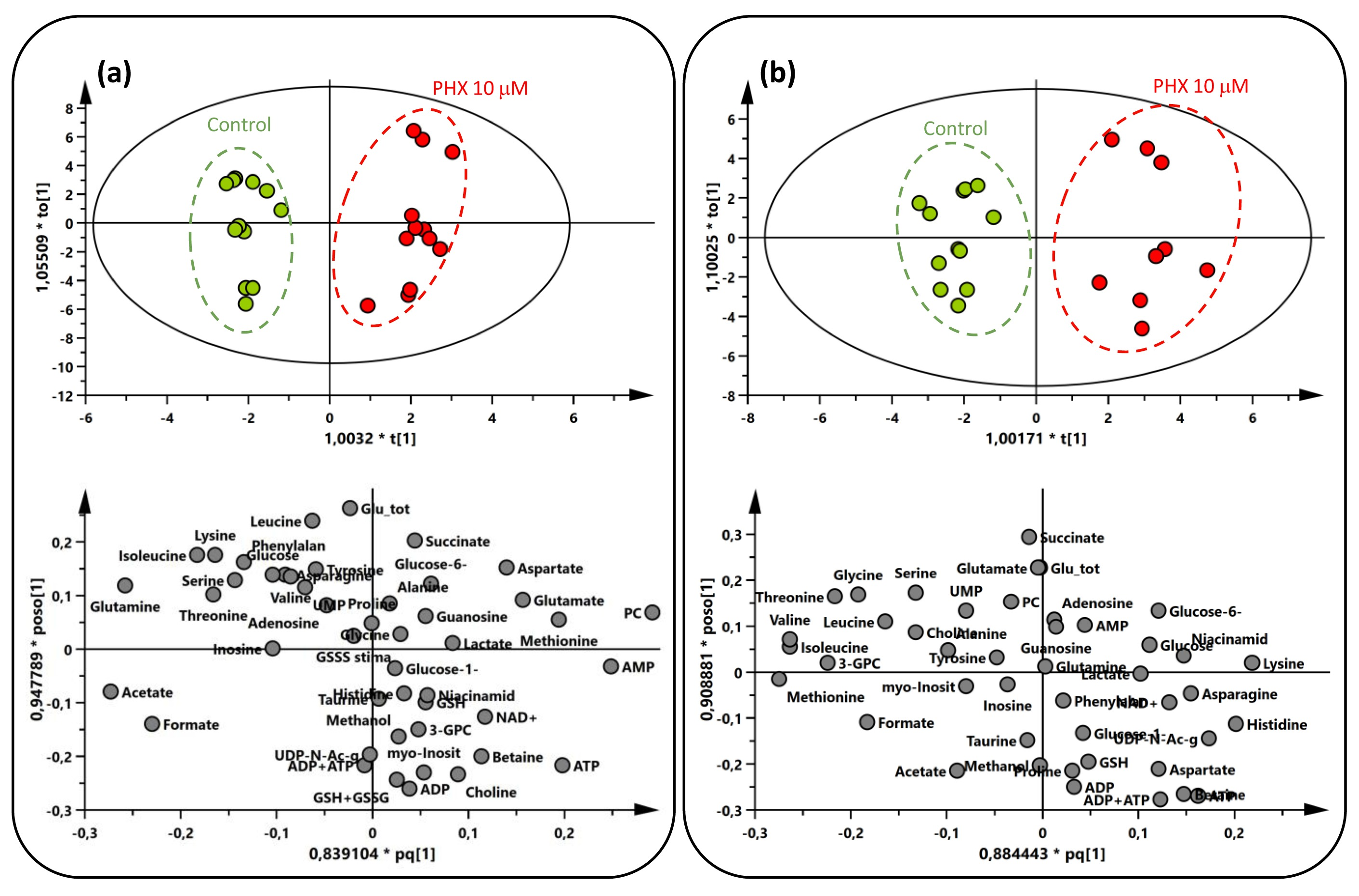

Supplement: S2 Fig — (a) OPLS-DA score plots of PHX (10μM) treated-sample vs control after 6 h N: 24; A: 1+2+0; R2X: 0.477; R2Y: 0.960; Q2: 0.943; CV Anova: 2.2E-09 and (b) 24 h N: 21; A: 1+1+0; R2X: 0.339; R2Y: 0.938; Q2: 0.827; CV Anova: 2.5E-05 (TIF) [file pntd.0008767.s002.tif]
