# Supplementary material for: Drug effects on metabolic profiles of Schistosoma mansoni adult male parasites detected by 1H-NMR spectroscopy
Source: PLoS Negl Trop Dis. 2020 Oct 12;14(10):e0008767. doi: 10.1371/journal.pntd.0008767 (PMC7580944; doi:10.1371/journal.pntd.0008767)
Supplement: S3 Fig — Networks are showed before (left panel) and after (right panel) neighbor-extension. Green and red colors represent respectively an increase or a decrease of the seed metabolite level in compound treated samples. Gray nodes represent “neighbor” metabolites. (PDF) [file pntd.0008767.s003.pdf]

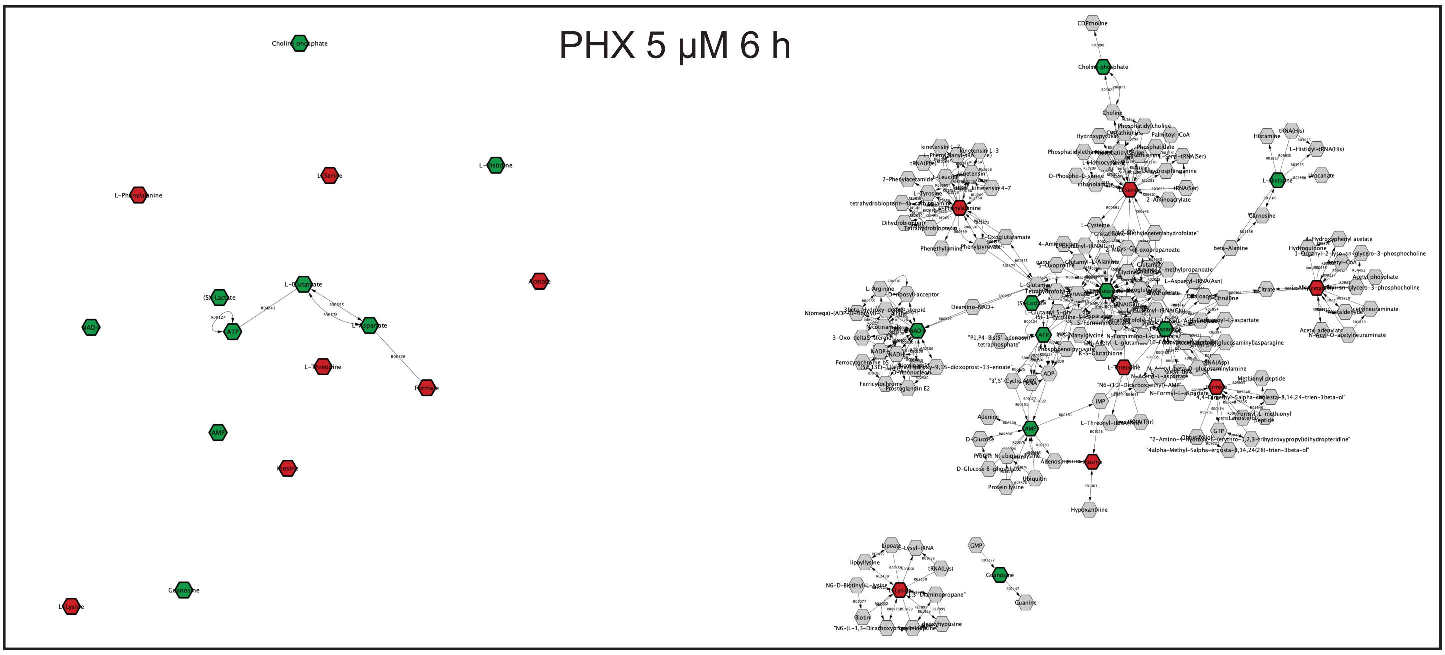
PHX 5 μM 6 h

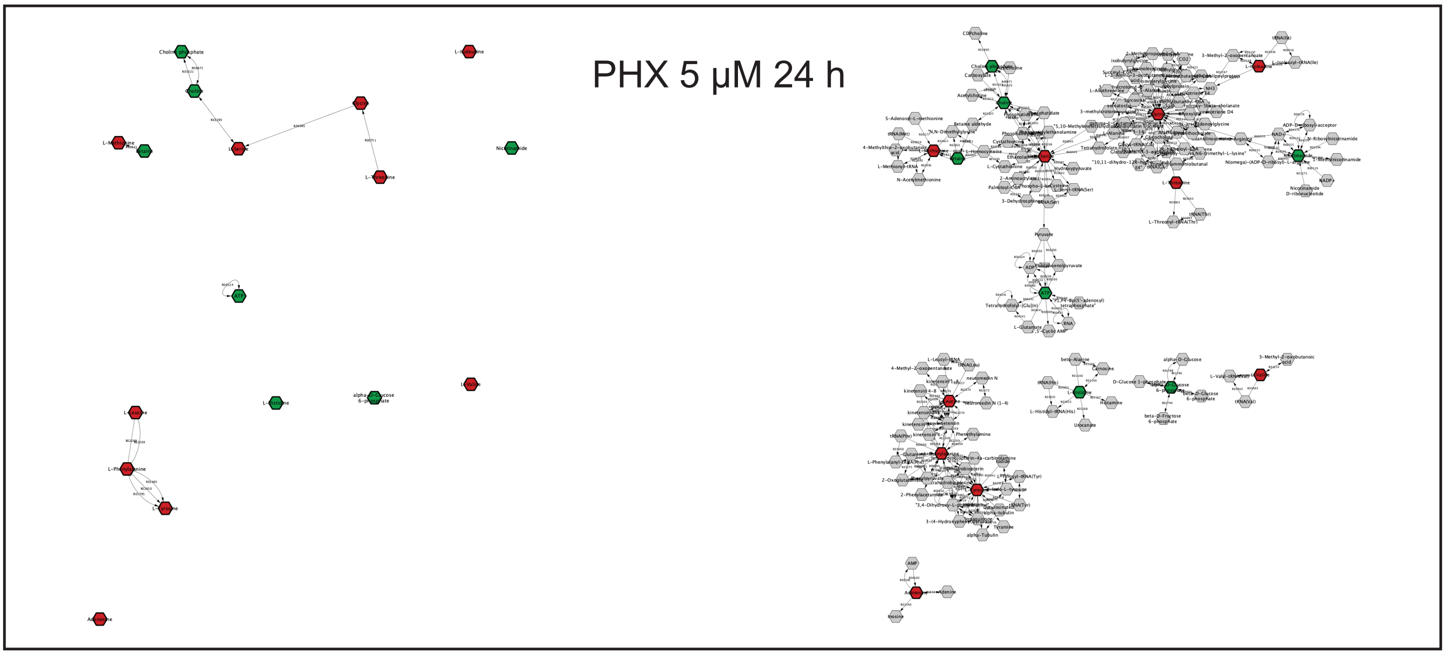
PHX 5 μM 24 h

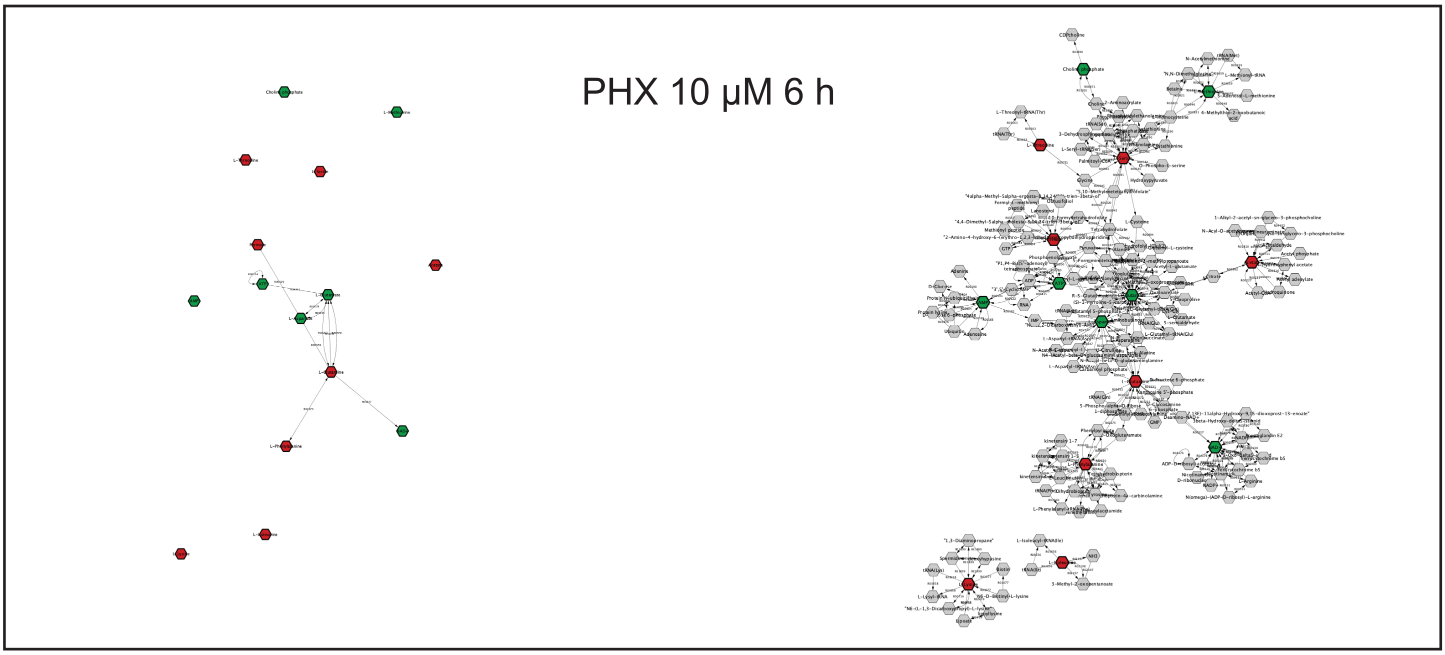
PHX 10 μM 6 h

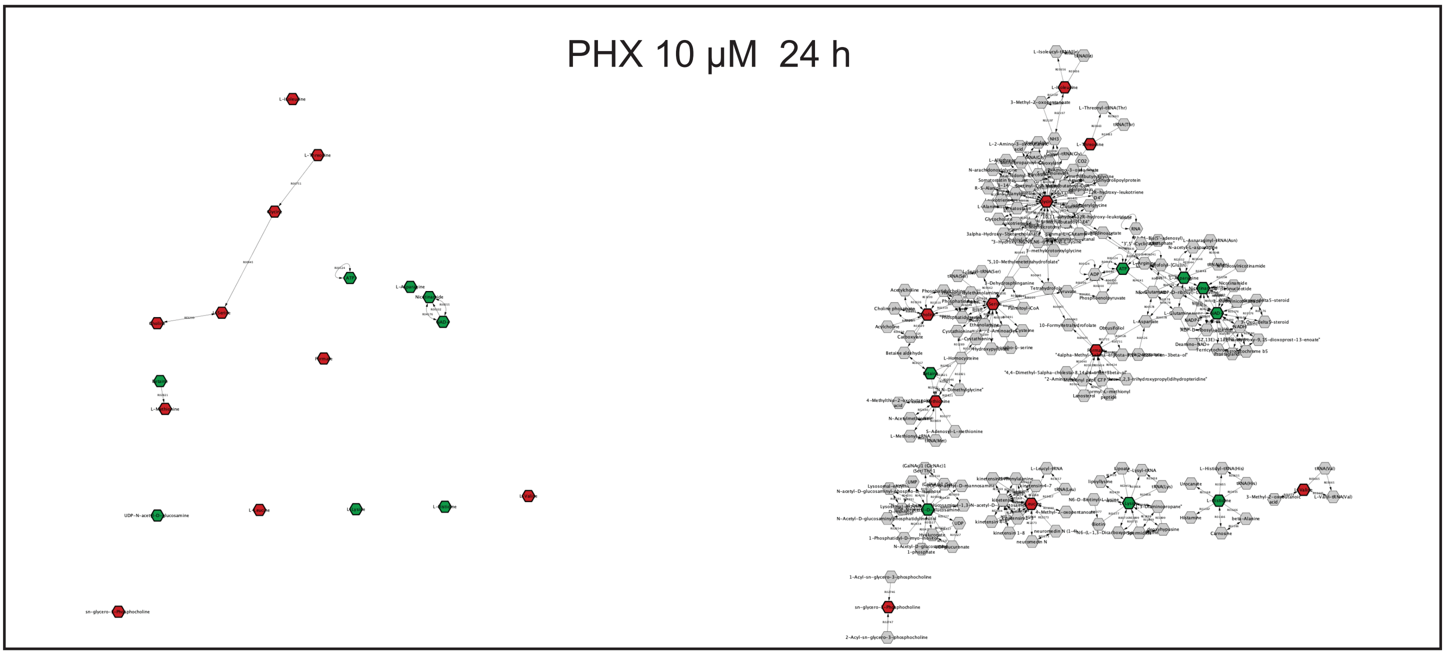
PHX 10 μM 24 h

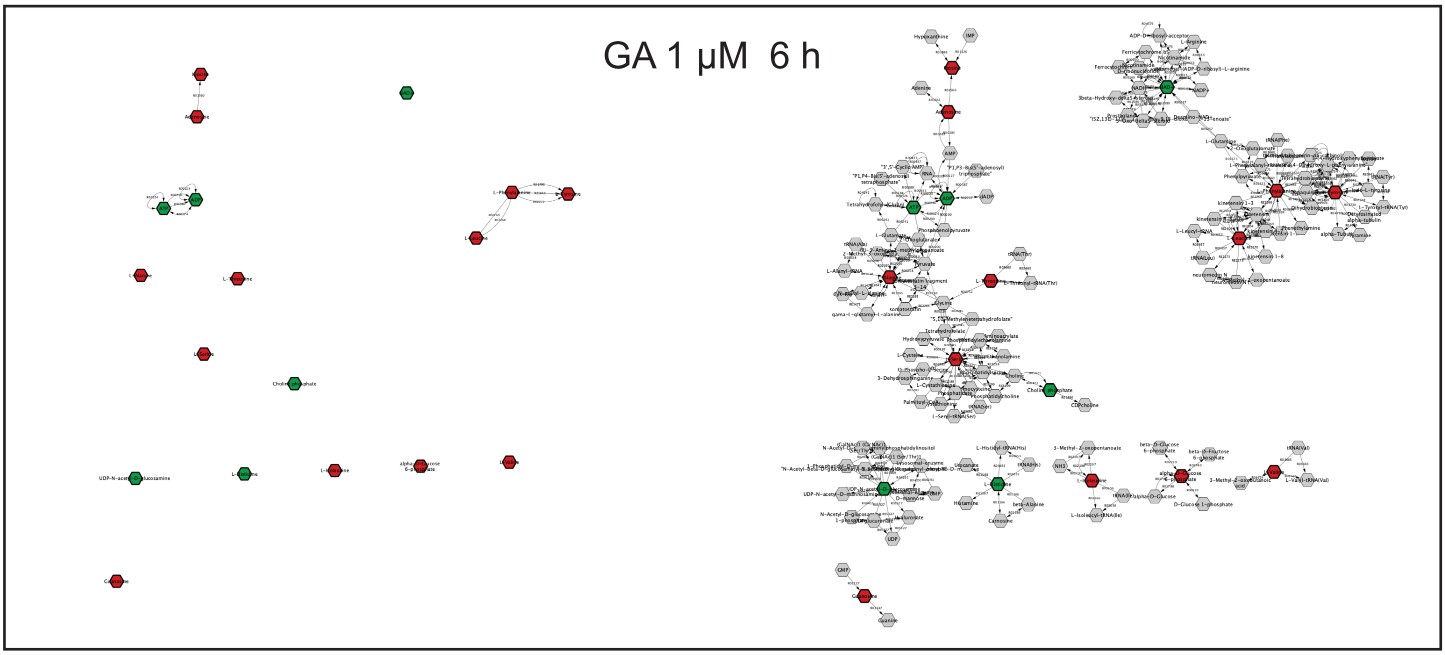
GA 1 μM 6 h

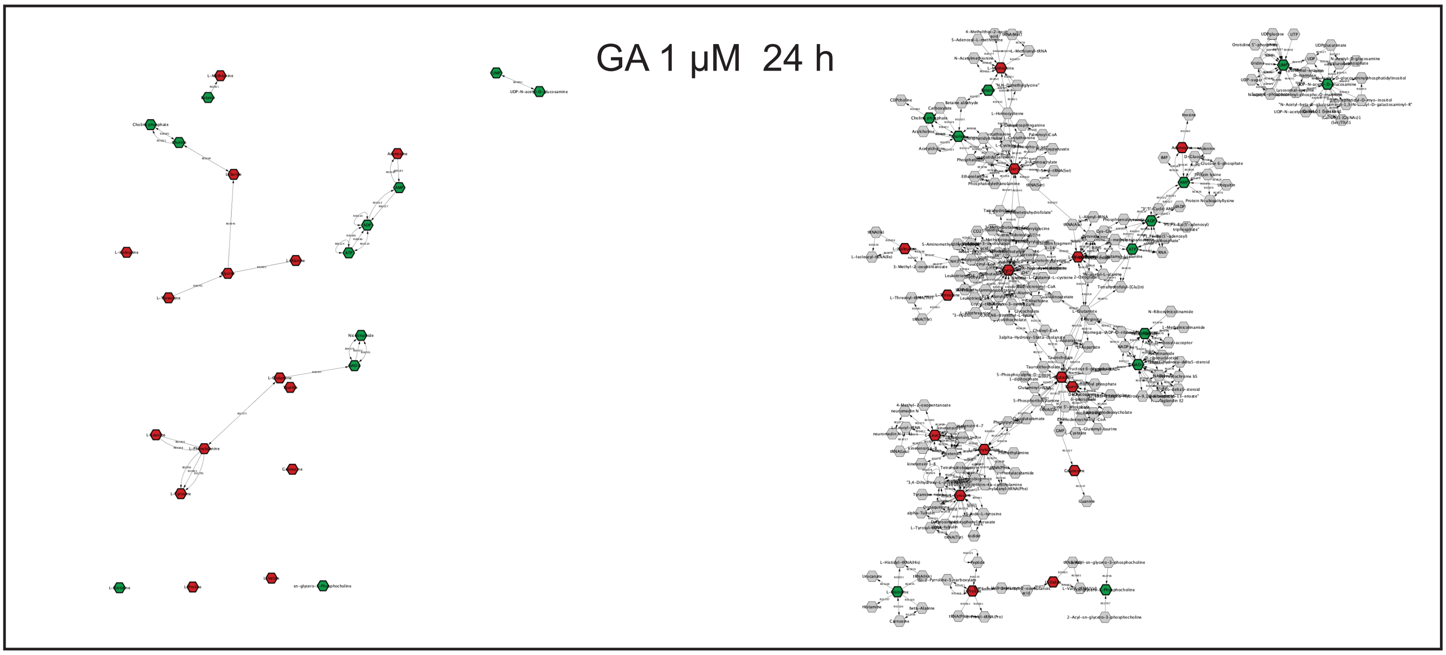
GA 1 μM 24 h
