# Supplementary material for: Drug effects on metabolic profiles of Schistosoma mansoni adult male parasites detected by 1H-NMR spectroscopy
Source: PLoS Negl Trop Dis. 2020 Oct 12;14(10):e0008767. doi: 10.1371/journal.pntd.0008767 (PMC7580944; doi:10.1371/journal.pntd.0008767)
Supplement: S1 Table — 1H and 13C chemical shifts are reported with respect to the TSP signal, and 31P chemical shifts are reported relative to 85% inorganic orthophosphoric acid. (DOCX) [file pntd.0008767.s004.docx]

**S1 Table. ^1^H, ^13^C and ^31^P assignments of metabolites identified in *Schistosoma* extract in H_2_O extracts**. ^1^H and ^13^C chemical shifts are reported with respect to the TSP signal, and ^31^P chemical shifts are reported relative to 85% inorganic orthophosphoric acid.

| **Compounds** | **Group** | **Assignment** | **^1^H [ppm]** | **^13^C [ppm]** | **^31^P [ppm]** |
| --- | --- | --- | --- | --- | --- |
| *Amino acids* |  |  |  |  |  |
| Alanine |  | α CH | 3.78 q | 53.5 |  |
|  |  | β CH_3_ | 1.48 d | 19.0 |  |
| Asparagine |  | α CH | 4.00 dd | 56.7 |  |
|  |  | β CH_2_ | 2.86 m; 2.96 m |  |  |
| Asparate |  | α CH | 3.89 dd |  |  |
|  |  | β CH_2_ | 2.82 dd; 2.67 dd |  |  |
| Glutamate |  | α CH | 3.76 q |  |  |
|  |  | β CH_2_ | 2.12 m; 2.08 m | 36.4 |  |
|  |  | γ CH_2_ | 2.67 m |  |  |
| Glutamine |  | α CH | 3.77 t | 57.0 |  |
|  |  | β CH_2_ | 2.14 m | 29.1 |  |
|  |  | γ CH_2_ | 2.47 m; 2.45 m | 33.7 |  |
| Glycine |  | α CH_2_ | 3.56 s | 55.9 |  |
| Histidine |  | 2’ CH | 7.81 d |  |  |
|  |  | 4’ CH | 7.06 d |  |  |
|  |  | α CH | 3.09 dd |  |  |
|  |  | β CH_2_ | 3.22 dd; 3.15 dd |  |  |
| Isoleucine |  | α CH | 3.67 d |  |  |
|  |  | β CH | 1.98 m |  |  |
|  |  | γ CH_3_ | 1.02 d |  |  |
|  |  | γ CH_2_ | 1.47 m; 1.35 m |  |  |
|  |  | δ CH_3_ | 0.94 t |  |  |
| Leucine |  | α CH | 3.73 m |  |  |
|  |  | β CH_2_ | 1.73 m |  |  |
|  |  | γ CH | 1.69 m |  |  |
|  |  | δ CH_3_ | 0.97 t | 24.9 |  |
| Lysine |  | α CH | 3.76 t |  |  |
|  |  | β CH_2_ | 1.90 m |  |  |
|  |  | γ CH_2_ | 1.50 m; 1.44 m |  |  |
|  |  | δ CH_2_ | 1.72 m |  |  |
|  |  | ε CH_2_ | 3.03 t |  |  |
| Methionine |  | α CH | 3.85 dd |  |  |
|  |  | β CH_2_ | 2.20 m; 2.13 m |  |  |
|  |  | γ CH_2_ | 2.65 t |  |  |
|  |  | δ CH_3_ | 2.12 s |  |  |
| Phenylalanine |  | 2’/6’ CH | 7.33 d |  |  |
|  |  | 4’ CH 3’/5’CH | 7.38 m |  |  |
|  |  |  | 7.43 m |  |  |
|  |  | α CH | 3.99 dd |  |  |
|  |  | β CH_2_ | 3.27 m; 3.13 m |  |  |
| Proline |  | α CH | 4.13 dd | 64.1 |  |
|  |  | β CH_2_ | 2.36 m; 2.03 m | 31.9 |  |
|  |  | γ CH_2_ | 2.01 m | 26.6 |  |
|  |  | δ CH_2_ | 3.41 dt; 3.34 dt | 49.0 |  |
| Serine |  | α CH | 3.84 dd | 63.4 |  |
|  |  | β CH_2_ | 3.98 dd; 3.93 dd | 76.2 |  |
| Threonine |  | α CH | 3.58 d | 68.9 |  |
|  |  | β CH | 4.26 m | 22.4 |  |
|  |  | γ CH_3_ | 1.33 d |  |  |
| Tyrosine |  | 3’/5’ CH | 6.90 m |  |  |
|  |  | 2’/6’ CH | 7.19 m |  |  |
|  |  | α CH | 3.94 dd |  |  |
|  |  | β CH_2_ | 3.17 dd; 3.06 dd |  |  |
| Valine |  | α CH | 3.61 d |  |  |
|  |  | β CH | 2.26 m |  |  |
|  |  | γ' CH_3_ | 0.99 d |  |  |
|  |  | γ CH_3_ | 1.05 d |  |  |
| *Glutathione* |  |  |  |  |  |
| GSH | Glutamyl | α CH | 3.78 d |  |  |
|  |  | β CH_2_ | 2.167 m | 29.2 |  |
|  |  | γ CH_2_ | 2.59 m | 34.3 |  |
|  | Cisteinyl | α CH | 4.57 dd |  |  |
|  |  | β CH_2_ | 2.95 dd; 2.97 dd |  |  |
|  | Glycil | α CH_2_ |  |  |  |
| GSSG | Glutamyl | α CH | 3.78 |  |  |
|  |  | β CH_2_ | 2.17 | 29.2 |  |
|  |  | γ CH_2_ | 2.57; 2.53 | 34.3 |  |
|  | Cisteinyl | α CH | 4.76 | 41.6 |  |
|  |  | β CH_2_ | 3.31; 2.98 |  |  |
|  | Glycil | α CH_2_ | 3.78 m | 46.3 |  |
| *Nucleotides and related compounds* | |  |  |  |  |
| Adenosine | Ribose | 1’ | 6.08 d |  |  |
|  |  | 2’ | 4.81 s |  |  |
|  |  | 3’ | 4.43 dd |  |  |
|  |  | 4’ | 4.28 q |  |  |
|  |  | *CH_2_*-OH | 3.91 dd; 3.83 dd |  |  |
|  | Adenine | 2 | 8.35 s |  |  |
|  |  | 8 | 8.25 s |  |  |
| AMP/ADP/ATP | Ribose | 1’ | 6.15 d |  |  |
|  |  | 2’ | 4.80 t |  |  |
|  |  | 3’ | 4.51 dd |  |  |
|  |  | 4’ | 4.38 dd |  | 3.45 |
|  |  | *CH_2_*-OH | 4.02 m | 66.4 |  |
|  | Adenine | 2 | 8.61 s |  |  |
|  |  | 8 | 8.25 s |  |  |
| NAD+ | Ribose | 1’ | 6.04 d |  |  |
|  |  | 2’ | 4.77 t |  |  |
|  |  | 3’ | 4.51 m |  |  |
|  |  | 4’ | 4.37 m |  |  |
|  | Nicotinamide | 2 | 9.34 s |  |  |
|  |  | 3 | 8.84 d |  |  |
|  |  | 4 | 8.18 m |  |  |
|  |  | 5 | 9.15 d |  |  |
|  | Adenine | 2 | 8.44 s |  |  |
|  |  | 8 | 8.18 s |  |  |
| *Carboxylates* |  |  |  |  |  |
| Acetate |  | CH_3_ | 1.92 s |  |  |
| Formate |  | CH | 8.46 s |  |  |
| Lactate |  | CH | 4.11 q | 71.4 |  |
|  |  | CH_3_ | 1.33 d | 23.0 |  |
| Succinate |  | CH_2_ | 2.41 s |  |  |
| *Lipid metabolism and osmolytes* | |  |  |  |  |
| 3-GPC |  | α CH_2_ | 3.90 m |  | -0.11 |
|  |  | β CH | 3.92 m | 73.5 |  |
|  |  | γ CH_2_ | 3.63 m | 64.9 |  |
|  |  | α’ (ΝCH_2_) | 3.68 m | 68.9 |  |
|  |  | β’ (ΟCH_2_) | 4.33 m | 62.4 |  |
|  |  | N CH_3_ | 3.21 s | 56.8 |  |
| Betaine |  | α CH_2_ | 3.91 s | 69.1 |  |
|  |  | N CH_3_ | 3.25 s | 56.2 |  |
| Choline |  | α CH_2_ | 3.49 dd |  |  |
|  |  | β CH_2_ | 4.05 dd |  |  |
|  |  | N CH_3_ | 3.19 s | 56.8 |  |
| Inositol |  | CH-1/3 | 3.54 dd | 74.0 |  |
|  |  | CH-2 | 4.06 t | 75.0 |  |
|  |  | CH-4/6 | 3.62 t | 75.3 |  |
|  |  | CH-5 | 3.28 t | 77.2 |  |
| O-Phosphocholine |  | α (ΝCH_2_) | 3.57 m |  | 3.26 |
|  |  | β (ΟCH_2_) | 4.12 m; 3.20 s |  |  |
| *Others* |  |  |  |  |  |
| Nicotinamide |  | β CH | 8.24 dd |  |  |
|  |  | β' CH | 8.94 s |  |  |
|  |  | γ CH | 7.60 m |  |  |
|  |  | δ CH | 8.72 dd |  |  |

***Experimental Section***

^1^H-^1^H TOCSY experiment was acquired at 600 MHz with spectral windows of 13.0 and 10.4 ppm, with carrier frequency at 4.7 ppm and using 4096 x 512 points and 8 transients. Water was suppressed using pre-saturation pulses.

^1^H-^13^C HSQC experiments were acquired with a spectral window of 15 ppm x 100 ppm (carrier frequencies at 4.85 and 46.5 ppm for the aliphatic region and 110 ppm for the aromatic region) using 2048 x 600 data points and 36 transients.

^1^H-^31^P HSQC experiment was acquired with a spectral window of 15 ppm x 10 ppm (carrier frequencies at 4.85 and 5.0 ppm) using 2048 x 64 data points and 36 transients.
